# A protocol for assessing bias and robustness of social network metrics using GPS based radio-telemetry data

**DOI:** 10.1186/s40462-024-00494-6

**Published:** 2024-08-06

**Authors:** Prabhleen Kaur, Simone Ciuti, Federico Ossi, Francesca Cagnacci, Nicolas Morellet, Anne Loison, Kamal Atmeh, Philip McLoughlin, Adele K. Reinking, Jeffrey L. Beck, Anna C. Ortega, Matthew Kauffman, Mark S. Boyce, Amy Haigh, Anna David, Laura L. Griffin, Kimberly Conteddu, Jane Faull, Michael Salter-Townshend

**Affiliations:** 1 School of Mathematics and Statistics, University College Dublin, Dublin, Ireland; 2 Laboratory of Wildlife Ecology and Behaviour, School of Biology and Environmental Sciences, University College Dublin, Dublin, Ireland; 3 Animal Ecology Unit, Research and Innovation Center (CRI), Fondazione Edmund Mach, San Michele all’Adige, Italy; 4 NBFC, National Biodiversity Future Center, 90133 Palermo, Italy; 5 INRAE, CEFS, Université de Toulouse, Castanet-Tolosan, 31326 France; 6 LTSER ZA PYRénées GARonne, Auzeville-Tolosane, 31320 France; 7 Alpine Ecology Laboratory, Savoie Mont Blanc University, Chambéry, France; 8 Biometrics and Evolutionary Biology Laboratory, Claude Bernard University Lyon 1, Lyon, France; 9 Department of Biology, University of Saskatchewan, Saskatoon, Canada; 10 Cooperative Institute for Research in the Atmosphere, Colorado State University, Fort Collins, USA; 11 Department of Ecosystem Science and Management, University of Wyoming, Laramie, USA; 12 Program in Ecology, University of Wyoming, Laramie, WY 82071 USA; 13 U.S. Geological Survey, Wyoming Cooperative Fish and Wildlife Research Unit, Laramie, USA; 14 Wyoming Cooperative Fish and Wildlife Research Unit, Department of Zoology and Physiology, University of Wyoming, Laramie, USA; 15 Department of Biological Sciences, University of Alberta, Edmonton, AB T6G 2R3 Canada; 16 Graduate Degree Program in Ecology, Colorado State University, Fort Collins, USA; 17 Department of Fish, Wildlife, and Conservation Biology, Colorado State University, Fort Collins, USA

**Keywords:** Bootstrapping, Correlation, GPS-based radiotelemetry, Network metrics, Permutations, Social network analysis, Sub-sampling, Uncertainty

## Abstract

**Background:**

Social network analysis of animal societies allows scientists to test hypotheses about social evolution, behaviour, and dynamic processes. However, the accuracy of estimated metrics depends on data characteristics like sample proportion, sample size, and frequency. A protocol is needed to assess for bias and robustness of social network metrics estimated for the animal populations especially when a limited number of individuals are monitored.

**Methods:**

We used GPS telemetry datasets of five ungulate species to combine known social network approaches with novel ones into a comprehensive five-step protocol. To quantify the bias and uncertainty in the network metrics obtained from a partial population, we presented novel statistical methods which are particularly suited for autocorrelated data, such as telemetry relocations. The protocol was validated using a sixth species, the fallow deer, with a known population size where $\sim 85\%$ of the individuals have been directly monitored.

**Results:**

Through the protocol, we demonstrated how pre-network data permutations allow researchers to assess non-random aspects of interactions within a population. The protocol assesses bias in global network metrics, obtains confidence intervals, and quantifies uncertainty of global and node-level network metrics based on the number of nodes in the network. We found that global network metrics like density remained robust even with a lowered sample size, while local network metrics like eigenvector centrality were unreliable for four of the species. The fallow deer network showed low uncertainty and bias even at lower sampling proportions, indicating the importance of a thoroughly sampled population while demonstrating the accuracy of our evaluation methods for smaller samples.

**Conclusions:**

The protocol allows researchers to analyse GPS-based radio-telemetry or other data to determine the reliability of social network metrics. The estimates enable the statistical comparison of networks under different conditions, such as analysing daily and seasonal changes in the density of a network. The methods can also guide methodological decisions in animal social network research, such as sampling design and allow more accurate ecological inferences from the available data. The R package aniSNA enables researchers to implement this workflow on their dataset, generating reliable inferences and guiding methodological decisions.

## Introduction

Social network analysis (SNA) has proven to be a valuable toolkit for biologists to understand diverse interactions among animal populations and their effect on the environment [19, 43, 48, 52, 75]. SNA also helps in understanding how environmental factors influence the structure of animal populations [1, 42, 49, 51] and informs how minor changes at the individual level propagate changes in the overall behaviour of the population [4, 31, 53, 66], which in turn contributes to informing epidemiological models, implementing customized measures for disease control, designing wildlife conservation policies, and resource allocation [24, 68, 69, 82, 83]. The term SNA is used to refer to the analysis of network data in the context of social interactions, for which there is an expanding set of statistical models and inferential procedures (see [65] for an introduction). Under this definition, the observed interactions between individuals are taken as fact and the goal is to summarise the data and make various inferences from it about the structure of the population of individuals and perhaps also of the behaviour of each individual. In SNA for animals, nodes typically depict individual animals within a population, and edges represent the relationships or interactions between them. Nodes can also represent diverse entities like groups, or locations, depending on the research objective. Meanwhile, edges can denote various relationships between nodes, including social interactions (such as grooming or aggression), spatial or temporal associations (like proximity or co-occurrence), or communication links (such as vocalizations or chemical cues) (see [30] for more details). In this paper, we present a structured step-by-step protocol to assess the reliability and robustness of the more commonly reported network metrics, as calculated from interactions data constructed from observations of individuals.

One of the fundamental requirements for performing SNA on animals is that a substantial proportion of individuals in the population be uniquely identified and observed for a sufficient period [30]. Global Positioning System (GPS) based telemetry technology have led to a significant boost in animal tracking and enabled wildlife ecologists to monitor and map minute details of animal movements, including those of highly cryptic species [12, 18, 56, 73, 79]. However, deploying GPS devices can be expensive both in terms of device cost [33] (from a few hundred to several thousand dollars each), and the costs related to captures, an operation that is manpower-hungry. The challenges include geographical constraints of some or all individuals in the population where capture methods do not work, personality traits of individuals as some individuals are capture-shy, and ethical and other issues raised by some stakeholders [6, 50]. Therefore, researchers are able to gather high-resolution and frequency data, albeit usually on a small subset of the entire population.

Inference for a large population from a limited sample of individuals has significant limitation, especially while analysing social networks [37, 41]. This is a concern as the relations among the members obtained from a sample of GPS-tracked individuals under-represent their complete set of relationships [19]. Furthermore, missing individuals from the sample may strongly influence the sampled individuals’ social measures [20]. Thus, relational data could be expected to respond more unreliably to sampling from a population than other data types [67]. The relational nature of network data also causes it to violate the assumptions of independence that underlie most parametric statistical tests [30] and creates an additional challenge in using a sample of individuals to make inferences about the population.

It is therefore crucial that researchers evaluating social networks constructed from satellite telemetry data have the proper tools and a structured protocol to assess the robustness of their data subject to data rarefaction and randomization, as both the collected data and analytical methods are prone to biases inflicted by specifics of sampling protocols [37, 71] or the species under study [75]. The frequency of telemetry sampling affects the accuracy of social network metrics as much as the percentage of the population sampled [37]. Networks constructed using a subset of a population are termed as partial networks [67]. The effect of using partial networks on the properties of individual metrics in animal social network analysis has been an active area of investigation [5, 19, 21, 22, 60, 67, 70, 71]. Previous research has primarily focused on the impact of missing nodes on social network structure, implying that node-level network metrics like degree and strength are relatively resilient to missing individuals [22], whereas global network statistics derived from partial data are biased estimators of overall network topology [7]. It nevertheless remains questionable how partial data affect the accuracy of inference obtained from the resultant networks, and how well the point estimates obtained from sampling represent the ecological processes occurring in a population. At this stage, standard methods for estimating the extent to which a partial network accurately depicts the underlying social structure [29], as well as the related level of uncertainty [8], must be developed.

Some preliminary work has been conducted to determine scaling methods; predict true network statistics from a partial knowledge of nodes, links, or weights of a network; and eventually validate the results on simulated networks and social media reply networks [7, 37]. A simulation study has further highlighted the importance of understanding consequences of missing random nodes from a complete animal social network [67]. Specifically, the networks have been simulated following a typical fluid fission fusion social system to determine the precision and accuracy of measures of individual social positions based on incomplete knowledge. On the contrary of what was expected, one of the findings of [67] revealed that in social networks based on fluid social interactions, precise inferences about individual social position can be derived even when not all individuals in a population are identifiable. A three part series [70–72] has examined the effect of random and non-random missing data on measurement bias and concluded that the bias varies considerably across scenarios, with the degree of bias being highly dependent on the metric of interest, the structure of the network being analysed, and the type of missing data. [72] also examined various imputation approaches for partial data and whether one should be used depending on the settings and network measurements. Despite the relevance of such discoveries and advancements in this field, social network analysis is being used in a limited way to study animal societies. Simulated data can be very useful for understanding the impact of missing data on the accuracy of inference obtained from partial networks. However, it has its limitations, as it may not fully reflect the nuances of empirical data or the practical challenges that ecologists face when dealing with incomplete observations from animals. Therefore, there is a need to understand the level of uncertainty [40] and associated bias in empirical data and also the extent to which the current methods adopted to estimate partial networks from sampled populations truly capture the underlying structure of animal social networks [30, 67, 76].

Our paper aims to present methods (both novel and already introduced by previous research) structured in a step-by-step protocol that can assess the adequacy(i.e., based on estimated bias and uncertainty) of the available data sample to perform social network analysis and obtain a measure of accuracy for global and node-level network metrics [30]. We demonstrate this by using data from free ranging animals, with their unique nuances, as an example. Our approach is particularly suited (but not limited) to telemetry relocations considering their autocorrelated structure [11], but we foresee that this can be used for observational data as well. For this, we present a five-step workflow applied to GPS telemetry observations of multiple species of ungulates with differing ecology and living in heterogeneous ecosystems.

1. The first step is to determine if the network structure obtained from the available sample of GPS observations captures any non-random aspects of the association in terms of the network metrics of interest. For this, we generate null networks by permuting a pre-network data stream. If a specific network metric does not meet this requirement, it should be discarded by researchers in their specific study case.
2. The second step is to assess how bias in the retained network summary statistics of interest varies with a decrease in the proportion of individuals sampled. Sub-sampling from the observed network helps estimate the extent of uncertainty in the network summary statistics and provides an idea of the robustness of the available sample.
3. The third step is to explore how different the network properties would have been if the researchers had tagged a completely different set of individuals from the population. This is achieved by applying a bootstrapping technique on the subsamples of the observed network. We also assess uncertainty by obtaining confidence intervals around the values of observed global network statistics, which is also critical when it comes to comparing networks (e.g., daily or seasonal changes in sociality, or between two populations of the same species).
4. The fourth step is to check how the node-level network metrics are affected by the proportion of individuals present in the sample. We use correlation and regression analyses to assess the robustness of node-level characteristics.
5. The final step in the workflow employs another bootstrapping approach to generate confidence intervals for each node’s individual network metric value, therefore generating node (individual) - level estimates (along with their uncertainty) supporting researchers to combine social connectivity of individuals (e.g., strength of their interactions with other individuals in the population) with other ecological parameters of interest (e.g., survival, mating strategy and success, habitat selection, and movement behaviour, to name a few).

Note that the last two steps examine the performance of node-level metrics and are not dependent on the first three steps and therefore, can be used at any point during the analysis. Using this step-by-step approach on a collection of observed GPS telemetry data assists in obtaining reliable statistical inference and provides improved conclusions from social network-based research for the use in ecological studies. We conclude our paper by outlining the methods described above and provide a stepwise protocol for ecologists on the application of these with their datasets. We also discuss some key considerations that should be made in both the construction and analysis of animal social networks [19, 47, 54, 80]. We have recently published a companion R software package aniSNA [45], which serves as a ready-made toolkit to apply the methods described in this paper in their animal social network studies. The package is built around the workflow described above and includes simple-to-implement functions which can be used directly with the observed set of GPS telemetry observations [46]. The package aniSNA performs the workflow with any choice of global or node-level network metric.

## Methods

### Data

We collated high-frequency GPS telemetry relocations’ datasets from five species of ungulates, including caribou (*Rangifer tarandus*), elk (*Cervus canadensis*), mule deer (*Odocoileus hemionus*), pronghorn (*Antilocapra americana*), and roe deer (*Capreolus capreolus*) belonging to four different geographical regions (Table 1). These datasets have been used and published in previous publications and we refer to them for details on animal captures, monitoring, and project goals (caribou: [78], elk: [16], mule deer: [58], pronghorn: [63], roe deer: [55]). These large datasets consisted of observations from an unknown proportion of individuals sampled from the population and contain a unique animal identity number, date, time, and spatial coordinates of the observations. Note that the reason for including multiple ungulate species with varying sampling designs, species ecology, monitoring periods and relocation rates was to illustrate that despite these differences, our protocol was able to capture robustness and bias of both global and node-level social network metrics, suggesting its applicability to a broader range of target species and ecological applications. To further demonstrate the validity of our workflow, we included a sixth dataset (presented in Analysis of fallow deer population in Phoenix Park), which is observational data collected from the Phoenix Park fallow deer *(Dama dama)* population in Dublin, Ireland [39]. Unlike the five datasets introduced earlier, the vast majority ($\sim 85\%$) of the population residing in Phoenix Park was observed and therefore allowed us to verify the validity of our approach in a special case where both the actual population size and the portion of it monitored by researchers was known.

**Table 1:** Summary of the data available for five species of ungulates monitored using satellite telemetry in North America and Europe

Species name	Area of observation	Centroid (Lat, Lon)	Number of animals observed	Total number of observations	Duration of observation	Fix rate
Caribou	Saskatchewan, Canada	(57.14489, − 104.3752)	94 (F:94, M:0)	304,607	03/2014 to 03/2018	Every 5 h
Elk	Rocky mountains, Alberta, Canada	(49.52496, − 114.3014)	171 (F:111,M:60)	856,241	01/2007 to 03/2013	Every 2 h
Mule Deer	Red Desert, Wyoming, US	(42.24222, − 109.2664)	263 (F:256, M:7)	1,458,043	03/2014 to 06/2021	Every 1–2 h
Pronghorn	Red Desert, Wyoming, US	(41.60466, − 107.9531 )	159 (F:159, M:0)	896,401	11/2013 to 10/2016	Every 2 h
Roe Deer	Aurignac, France	(43.28552, 0.8809104)	147 (F:81,M:66)	419,165	01/2005 to 12/2012	Every 10 min

In the following section we described how we computed individual association and the related network, followed by a detailed description of our five-step protocol.

### Identifying associations and network construction

We obtained network structure from the raw data stream by identifying associations between each pair. We considered a pair of individuals in the sample to be associating if the two animals were observed within *s* metres from each other and within a time frame of *t* minutes. The value of spatial threshold *s* can be chosen by applying a statistical approach to the observed data. He et al. [41] suggest one such approach could be to use the first mode from the distribution of inter-individual distances as it likely represents socially associating individuals. The temporal threshold *t* is dictated by the fix rates in telemetry data. For example, GPS collars on animals send signals consisting of spatial coordinates after a predetermined time interval. These signals can be received a few seconds (up to a few minutes) before or after the expected time. Therefore, temporal thresholds should be chosen in such a way that it accounts for this flexibility. See Sensitivity analysis for temporal threshold for sensitivity analysis on the choice of temporal threshold. Researchers should generally pick a threshold based on their device accuracy, species ecology, and research question. To get a more accurate set of interactions, the approach introduced by [84] for generating interaction networks can be implemented as Step 0 of our protocol. The method relies on continuous-time movement models [13] that enable researchers to recover underlying interactions that could not be observed directly.

We calculated an association index through a modified version of Simple Ratio Index [30] for GPS telemetry observations. He et al. [41] argue that for GPS data, an observation of individual A without individual B is only informative if B is observed elsewhere simultaneously. Therefore, the denominator of the original formula should only include observations where GPS data are simultaneously available for both individuals. The modified index used in the analysis is as follows:

$$\begin{aligned} \text {Index} = \frac{x_{AB}}{x_{AB} + y_{AB}} \end{aligned}$$

where $x_{AB} -$ No. of times when A and B are observed associating, $y_{AB} -$No. of times A and B are observed within the temporal threshold but not associating.

The value of the index can range between 0 and 1, where 0 would indicate that the two animals were never observed together and 1 that they were always observed together. The individuals sampled from the population form the network nodes, and an association between pairs accounts for the edges in the networks. Each edge in the network has a weight attribute that reflects the association strength calculated by the modified Simple Ratio Index described above. In this way, we obtained the network structures corresponding to each species from their raw GPS observations, which represented the complete set of relationships among the individuals tagged for each species. For the five-step workflow, researchers have the flexibility to create custom indices tailored to their specific research objectives for calculating the association between pairs of animals. These indices can be adjusted to account for both the distance between the animals during each observation and the duration of each contact [15, 30].

### Analysis

To assess the properties of the networks, we used standard metrics common in animal social network analysis (Table 2). The local network summary statistics which provided individual-level information included degree [66], strength [61], betweenness centrality [4, 44], eigenvector centrality [4, 52] and local clustering coefficient [61, 66]. Edge density [59], transitivity, and diameter are the global network metrics and provide a summary of the overall network and behaviour of the individuals as a whole. We also calculated the mean of each node’s degree (mean degree [66]) and strength (mean strength) [76] and used those as global network properties.

**Table 2:** Network metrics used in the analyses

Metric	Type	What does the metric measure ?
Degree	Node-level	The number of connections an individual has in the network. Higher degree means more gregariousness
Strength (weighted degree)	Node-level	The combined weight (i.e., frequency or duration) of all of an individual’s connections in a network. It indicates the level of social connectivity based on the intensity of interactions among individuals
Betweenness centrality	Node-level	The number of times an individual occurs on the shortest path between two other individuals in the network. Betweenness indicates the importance of an individual to act as a bridge, quantifying its influence over the flow of information or interactions between other individuals in the network
Eigenvector centrality	Node-level	A measure of influence in the network that takes into account second-order connections. Eigenvector centrality of a node indicates its influence within the network based on the quality of its connections, considering the centrality of its neighbours
Local clustering coefficient	Node-level	A measure of likelihood that the connections of an individual are also connected. It reflects the level of clustering or cohesion within a node’s immediate social connections
Edge density	Global	The proportion of completed edges in the network. It indicates the overall level of connectivity withing the network, reflecting the extent to which individuals are interconnected through social interaction
Transitivity	Global	The amount of clustering in the network, calculated as a function of completed triangles relative to possible triangles. It reflects the degree of reciprocity in social interactions within the network
Diameter	Global	The shortest distance between the two most distant individuals in the network. It provides insight into the extent of social connectivity and potential pathways of information transmission
Mean degree	Global	Average number of connections of an individual in the network. It provides insight into the overall level of social engagement within the population
Mean strength	Global	Average strength of an individual in the network. It indicates the average level of social strength within a network

#### Step 1. Pre-network data permutations

We generated null models to assess whether the interactions captured by the observed sample were genuinely caused by social preferences, as opposed to random associations. Null models were constructed to account for non-social factors that led to the co-occurrence of animals. In animal social network analysis, null models are broadly classified in two ways: network permutations and pre-network permutations [27]. Network permutations are performed after the network is generated from the data, whereas pre-network permutations are performed on the data stream before generating networks from it. High resolution GPS telemetry observations often generate data in the form of autocorrelated streams where the extent of autocorrelation depends on the speed of the individual compared to time resolution. In the permuted versions of the data, we wanted to maintain this autocorrelation structure (if present) of each individual’s movements but randomise the contacts with other individuals. Therefore, we obtained pre-network datastream permutations as suggested by [27, 77]. For each individual in the study, we segmented the tracks walked each day. Then the dates on which those tracks were followed were shuffled for each individual. This methodology of permuting the pre-network data stream ensured unaffected home ranges of animals in the permuted data, but whom they came in contact with was now randomised in the null model. This also preserved the autocorrelated structure of individual tracks to ensure realistic animal movements.

For each species, we obtained 1000 permuted versions of the raw data stream, giving rise to 1000 network structures. Then, we calculated global network summary statistics of density, mean strength, transitivity, and diameter for each of those networks and obtained a null distribution of values. Analysing the relative position of the observed network metric’s point estimate with respect to the distribution of null values helped determine the metrics that capture non-random aspects of the observed network.

#### Step 2. Sub-sampling from the observed network

We randomly sub-sampled *m* nodes from the observed network of *N* nodes where $m<N$ without replacement. All the associations among the sampled nodes were preserved, and the rest were dropped. This resulted in a network structure that would have been obtained if originally just these *m* individuals had been tagged from the population. In this way, we drew 100 samples of size *m* where the value of *m* ranged from 10% to 90% of the total nodes forming each network for five species. We recorded the values of global network metrics of density, mean strength, transitivity, and diameter and obtained a distribution of the values. We assessed the bias in the values of network metrics obtained from this sub-network compared to the original network. Performing this procedure across five species and for different values of *m* would reveal which network metrics are robust to sub-sampling and should be adopted for social network studies on the target species and available samples.

We also applied a sub-sampling approach on the permuted networks to determine under what sampling level the observed networks resembled the random networks. We sub-sampled nodes from 1000 permuted network versions without replacement at different levels ranging from 10% to 90%. We calculated four global network metrics for each permuted version, and each level and their distribution were plotted along with the distribution of sub-sampled versions of the original network. This visualisation was aimed to provide an estimate of the minimum amount of subsampling required to ensure that the network differed significantly from a random network for that species and the associated environment.

#### Step 3. Bootstrapped confidence intervals for global network metrics

Assuming a researcher has chosen a set of network metrics appropriately, it would be prudent to consider not only the point estimate derived from their data but also the uncertainty associated with it. To create confidence intervals to facilitate the comparison of different networks (e.g., differing sampled individuals from the same population such as those living in areas disturbed by humans vs those that were not, or the same individuals’ networks computed at different times), we adapted the bootstrap algorithms of [74]. Similar to the algorithms used in SOCPROG [81] and UCINET [10], this algorithm sampled nodes in the network with replacement for each of B=1000 bootstrap replications. Each bootstrap replication network comprised the same number of nodes (animals) as the original network; however, some of the original nodes were absent, some were present once, and some more than once. In each replication, edges between any two different sampled nodes were retained, whereas edges between the same node resampled twice were randomly chosen from the set of all original edges. Bootstrapping has been used to infer uncertainty in animal social networks (see [54, 80]), however bootstrapping social network data should only be used carefully as zero edges (which could result from unobserved associations rather than two animals not associating at all) are resampled as zeros across all replications [28]. We, therefore, assessed whether such algorithms were appropriate for constructing confidence intervals for network metrics. See Assessment of bootstrapping algorithm for details on the assessment of our bootstrapping algorithm, including the results showing correct calibration under the null hypothesis.

We examined how uncertainty was related to sample size and obtained confidence intervals using the bootstrapped samples for each global network metric of density, mean strength, transitivity, and diameter for different sample sizes. We recorded the width of confidence intervals for each network metric corresponding to different sample sizes. Finally, we repeated this process ten times and calculated the mean width of the ten confidence intervals against the number of individuals in the network. This provided the level of uncertainty associated with a particular network metric for a given amount of data.

#### Step 4. Correlation analysis between node-level metrics of partial and full networks

To assess the accuracy of the node-level metrics inferred from a given sample, we evaluated the correlation between the values of the metrics in the observed sample, and a smaller sub-sample of the empirical data as suggested by [37, 67]. First, we calculated node-level metrics of degree, strength, betweenness, clustering coefficient, and eigenvector centrality for each node in the observed network. Then we sub-sampled nodes from the observed network at 10%, 30%, 50%, 70%, and 90% levels without replacement and calculated node-level metrics for each sub-sample. Finally, we calculated the correlation coefficient between the metric values of the nodes in the observed and partial networks. The process was repeated 10 times at each level of sub-sampling, and the mean and the standard deviation were recorded from the 10 correlation coefficients at each level. We also ran a regression analysis [67] to assess how the values of node-level metrics for partial networks relate to their values in the whole network (See Regression analysis between node-level metrics of sub-sampled and observed networks).

#### Step 5. Bootstrapped confidence intervals for node-level network metrics

To obtain confidence intervals around the node-level network metrics [25], we obtained 1000 bootstrapped versions of the network (as described in Step 3 of the workflow) for each species. In the bootstrapped versions, nodes were sampled with replacement with implications for the metric values of each node. If a node (e.g., A) had 5 neighbours in the observed network, then in a bootstrapped version, node A could have had more or less than or equal to 5 neighbours depending on what other nodes were chosen in that sample and how many times. This method enabled us to create a distribution of metric values for node A that helped us understand how the choice of other nodes in the sample affected the observed metric value of node A.

We applied our workflow’s last four steps of subsample analysis, confidence intervals for global and node-level metrics as well as correlation analysis on the fallow deer population of Phoenix Park, Dublin. The first step of our five steps protocol was omitted for analysing observational data of fallow deer, as it is only needed for GPS telemetry observations, where an arbitrary distance threshold is used to define two individuals’ proximity. However, observational data already includes information about the group affiliations of each animal, and interactions occurring among them, making this initial step redundant. We therefore started with the second step, computing network metrics, and proceeded with the following steps of our protocol (See Analysis of fallow deer population in Phoenix Park for detailed analysis).

The protocol discussed above can also be applied to data collected through other techniques. Establishing a network structure involves compiling an edge list containing information on interactions between pairs of animals. Data collection techniques such as proximity loggers and camera traps can also serve as efficient technologies for gathering the necessary data to construct such an edge list and subsequently form a network structure. Proximity loggers, when affixed to animals, provide data on instances when individuals come into close proximity to one another [84]. Camera traps offer spatial insights into interactions among two or more animals [14]. These eliminate the need for imposing arbitrary spatial and temporal thresholds, as they inherently capture all genuine interactions. We conducted all analyses using R 4.1.3 [62] and the methods discussed as a part of our five step workflow are available as functions in the R software package aniSNA [45, 46].

## Results

### Association index and network formation

**Fig. 1:**
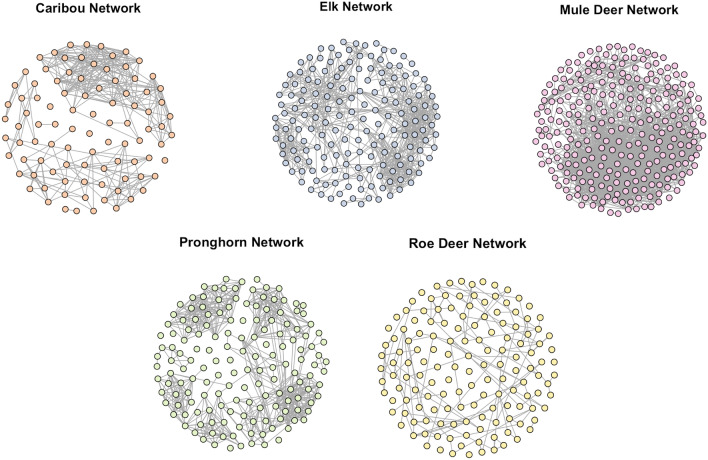
Network structures for the five large herbivores analysed in this study. Each node is an animal, and an edge between two nodes indicates that they have interacted at least once. Note how the pronghorn network is denser than the roe deer network despite having a very similar number of nodes. Also, note the clear partition in the caribou network

The spatial threshold was 10 m for mule deer sample and 15 m for all other species data. The temporal threshold was arbitrarily chosen to be 7 min, which accounted for delays in signal reception by the GPS devices. For example, if a GPS unit recorded a location at 09:57 AM, the observations recorded until 10:04 AM were evaluated for potential interactions. Table 3 shows the values of network summary statistics for each of the five species. Note that each of these networks had distinct characteristics. Despite having a lower spatial threshold value, the mule deer sample captured a substantial number of interactions, as indicated by its high mean degree and mean strength values. The elk sample had the maximum diameter with a value of 9 which implied that it took a maximum of 9 steps to reach from any individual to another in the elk network. The pronghorn sample had high transitivity and mean local clustering coefficient, indicating that any two associates of a pronghorn were likely to be associated with each other. The values in table 3 are indicative of the variation in different datasets included in the study and in any scenario, these networks should not be compared to one another in terms of metric values as the data collection was not standardised across different species. Figure 1 shows the network structures obtained for all five species.

**Table 3:** Summary statistics for the networks obtained from the five species

Species name	Order (nodes)	Size (edges)	Mean degree	Mean strength	Mean betweenness	Mean eigenvector centrality	Mean local clustering coefficient	Density	Transitivity	Diameter
Caribou	94	309	6.57	0.048	32.95	0.021	0.64	0.070	0.576	8
Elk	171	696	8.14	0.028	147.38	0.042	0.65	0.047	0.555	9
Mule Deer	263	1582	12.03	0.767	367.78	0.014	0.48	0.045	0.353	8
Pronghorn	159	525	6.60	0.023	34.32	0.021	0.67	0.042	0.636	7
Roe Deer	147	130	1.77	0.020	4.86	0.017	0.59	0.012	0.479	6

Order of a network represents the number of individuals tagged in the sample with mule deer sample having the greatest order and caribou sample having the smallest order

#### Step 1. Pre-network data permutations

**Fig. 2:**
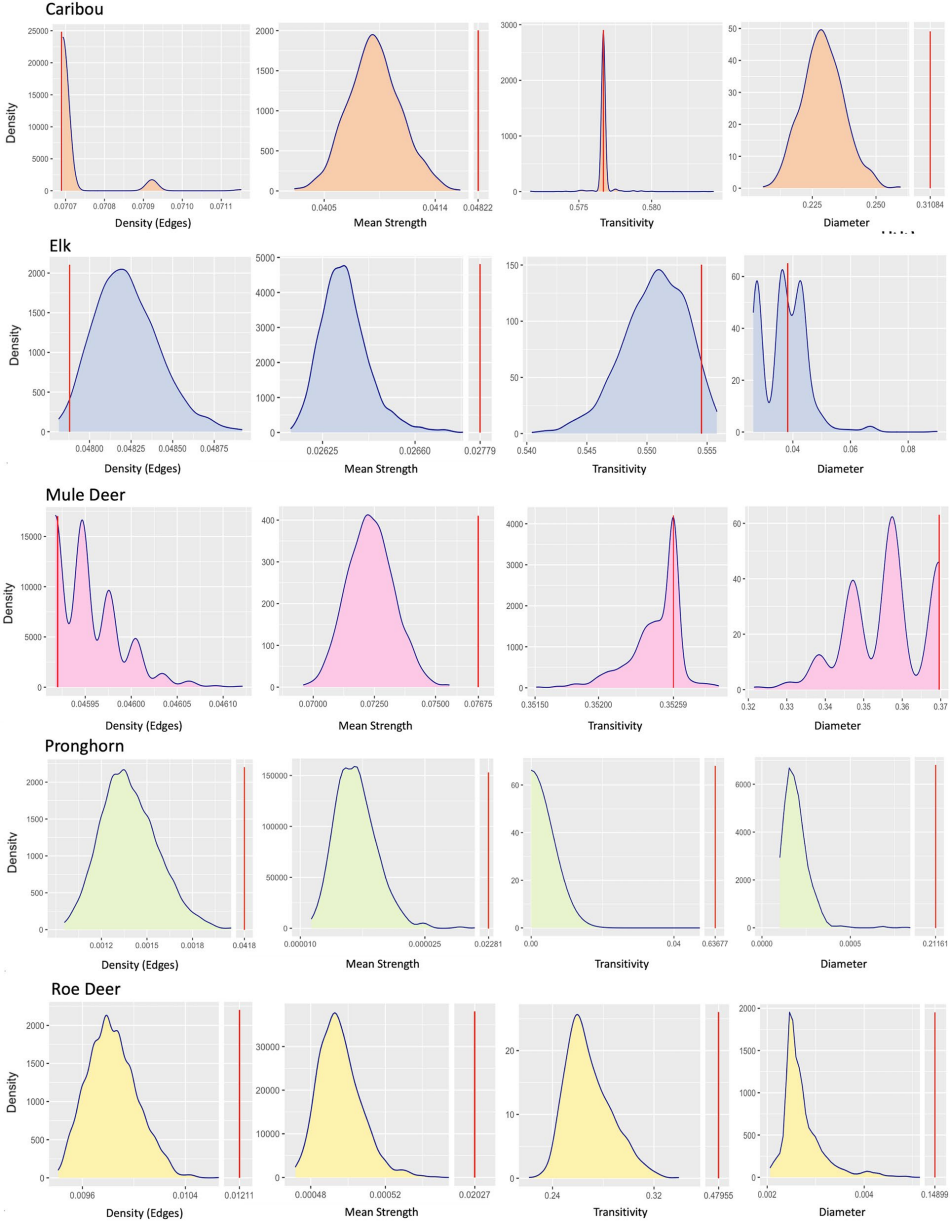
Rows correspond to the five species, while columns correspond to four standard network metrics. Each plot represents the distribution of network metric values obtained from 1000 permuted versions of the species network. The red bar in each plot represents the position of observed network’s metric value. For all five species, the observed value of mean strength was higher than the mean strength values from the permuted versions, indicating that all the samples successfully capture non-random interactions between the individuals. The network metrics whose observed value lie within the null distributed values indicate that those metrics are not different from a randomly generated network, such as the density and transitivity of caribou

For pronghorn and roe deer samples, the observed values were significantly different from the distribution of permuted network values (Fig. 2), indicating that these two samples captured non-random aspects of the population very well. Also, for all five species, the mean strength of the observed network was higher than the permuted networks (Fig. 2). This indicated that all these samples captured higher association rates than would be expected from a random network under similar assumptions. For caribou, elk, and mule deer, the observed value of transitivity was within the distribution of null values. In general, researchers should avoid making inference using those network metrics on data whose observed values lie within the distribution of null values. This is because, in that case, the network structure obtained from the available sample does not capture the non-random aspects of interaction concerning that particular network metric. To effectively demonstrate the remaining four steps of the protocol, we opted to analyze all five network metrics. This has allowed us to showcase the practical applicability of each step in the protocol and provided a thorough understanding of how each metric operates within the context of each species’ social network. Through this comprehensive demonstration, we have highlighted the versatility and effectiveness of the protocol in elucidating various aspects of social network analysis across different species.

#### Step 2. Sub-sampling from the observed network

**Fig. 3:**
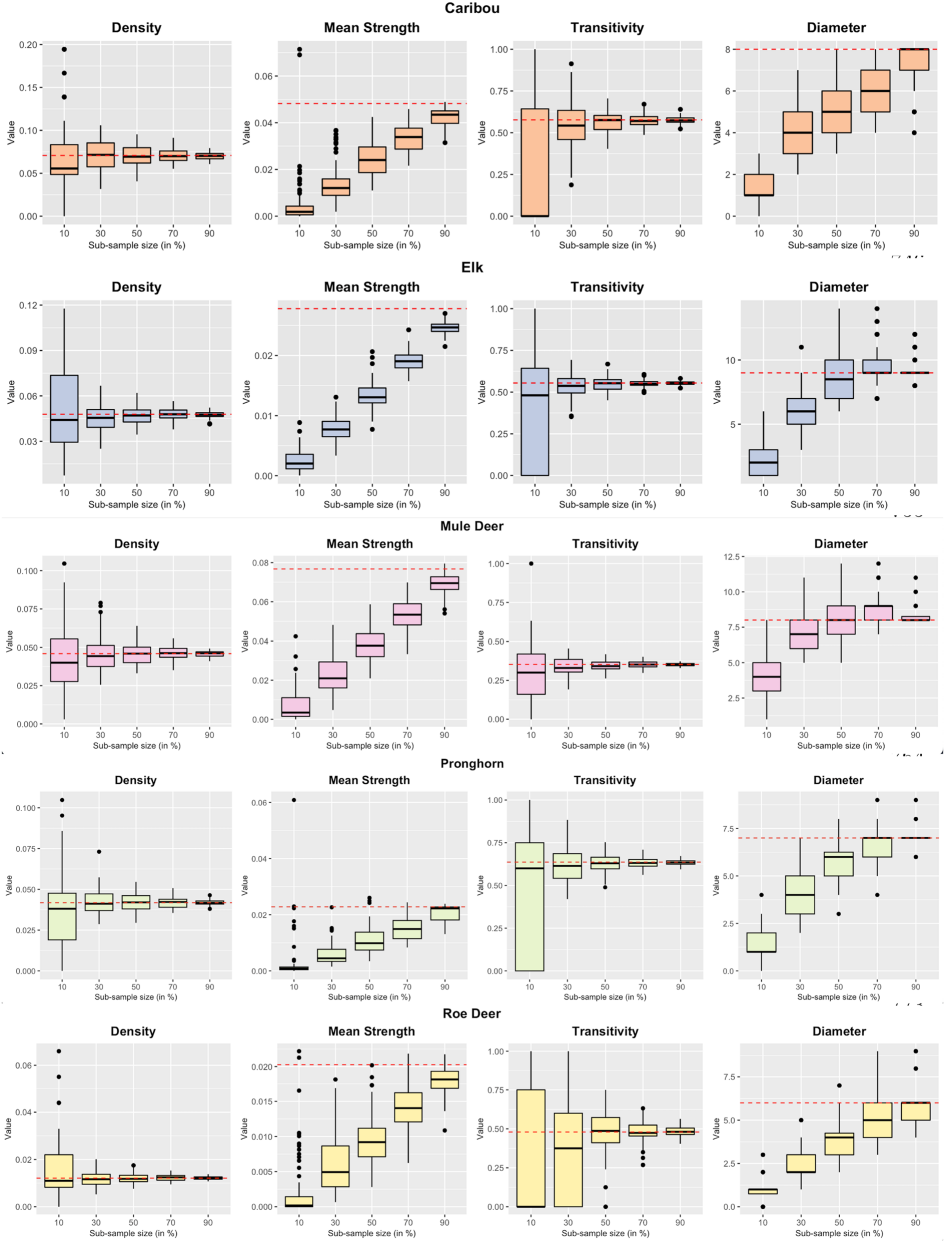
Effect of sub-sampling on four global network metrics. The horizontal red line in each plot represents the metric value in the observed network. The boxplots denote the distribution of network metric values obtained from the observed networks by taking 100 sub-samples at each sampling level. The size of the boxes in the boxplots of a network metric with respect to the sample size represents the extent of uncertainty in that network metric. For example, density has smaller boxes when as low as 10% of the nodes are selected. In contrast, the box size for transitivity is large, representing that density is more stable than transitivity. The extent of deviation of the box position from the horizontal red line depicts the bias in the calculated values of the network metric. The values of mean strength become biased as the sample sizes are lowered. This is because mean strength values are directly affected by the number of nodes present in the network

Performing sub-sampling on the observed networks of five species at various levels revealed the network metrics density and transitivity as the most stable and unbiased (Fig. 3). The uncertainty in these two metrics was comparatively low for four out of the five species, even when just 30% of the individuals were present in the sub-sample. Their distribution was centered on the true values, and this allowed us to estimate bias if the proportion of sampled individuals were known. Transitivity at a sub-sampling level of 10%, 10%, and 30% became unreliable for caribou, pronghorn, and roe deer respectively, implying that this metric was a poor measure when the sampling proportion was very small. The bias in mean strength values followed a linear pattern when the sub-sampled proportion was reduced from 90% to 10% for all five species. The linear pattern suggested that the bias for mean strength can be corrected if the sampling proportion was known. This linear increase must, however, plateau as we approach a census of the population [7]. The network’s diameter followed a staircase pattern with lowering sub-sampling levels but it was not linear for all five species and tended to plateau. Diameter and mean strength were directly affected by the number of nodes present in the sample. Therefore, care should be taken while using these metrics when the sampling proportion is unknown.

**Fig. 4:**
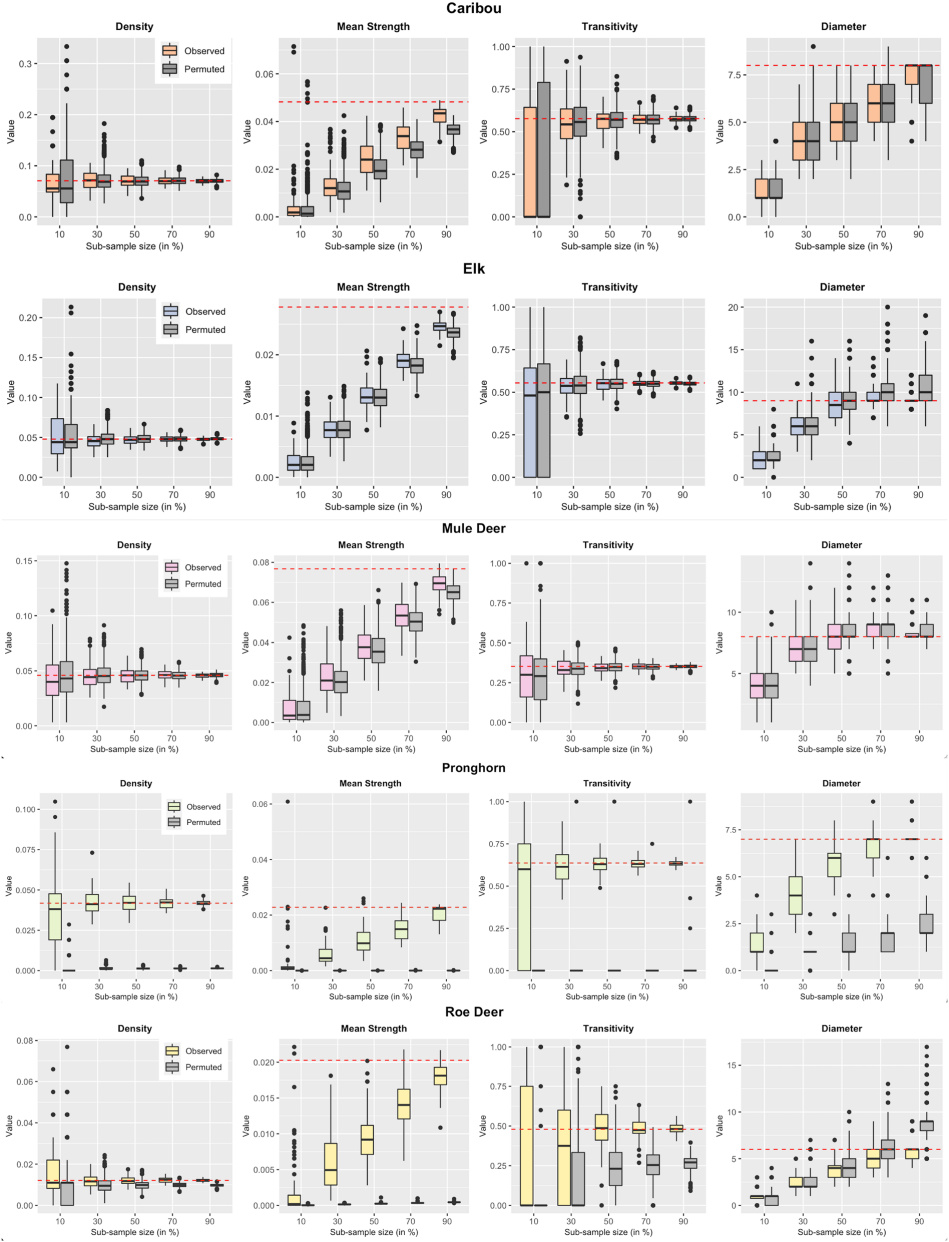
Sub-sampling of permuted networks. The grey boxplots are obtained by calculating network metric values on 1000 permuted versions. The non-grey boxplots are the ones that we obtained in Fig. 3. The horizontal red line in each plot represents the observed metric value. Comparing the subsamples of the observed network with those of permuted networks identifies the sample proportion where the non-random aspects of the observed network start looking similar to those of random networks. For example, the mean strength of caribou subsamples in the observed network starts to overlap with the distribution of permuted subsamples at 70% level and becomes almost identical at 10% level. On the other hand, the mean strength distribution of pronghorn subsamples remains higher than those of permuted subsamples distribution at as low as 10% level

We performed subsampling on 1000 permuted versions of the network along with the subsampling on the observed network. The side-by-side visualisation of the network metrics distribution (Fig. 4) enabled us to identify the sampling level at which a subsampled network began to resemble a random network and we can no longer make correct inference about an ecologically relevant hypothesis. The plots revealed that for caribou, elk, and mule deer, network metrics density, transitivity, and diameter distribution became identical to that of a null network at 90%, 90%, and 70% subsampling levels respectively. Nevertheless, mean strength distribution increasingly overlapped with the distribution of subsamples from the null network when the level of sub-sampling was lower than 90%. For pronghorn, the distribution of all the network metrics obtained from subsamples of the observed network was higher than the values obtained from the subsamples of the null networks at all sampling levels. For roe deer, this was only true for mean strength. The distribution of density and transitivity values overlapped the null distribution at 50% and 30% levels, respectively.

#### Step 3. Bootstrapped confidence intervals for global network metrics

**Fig. 5:**
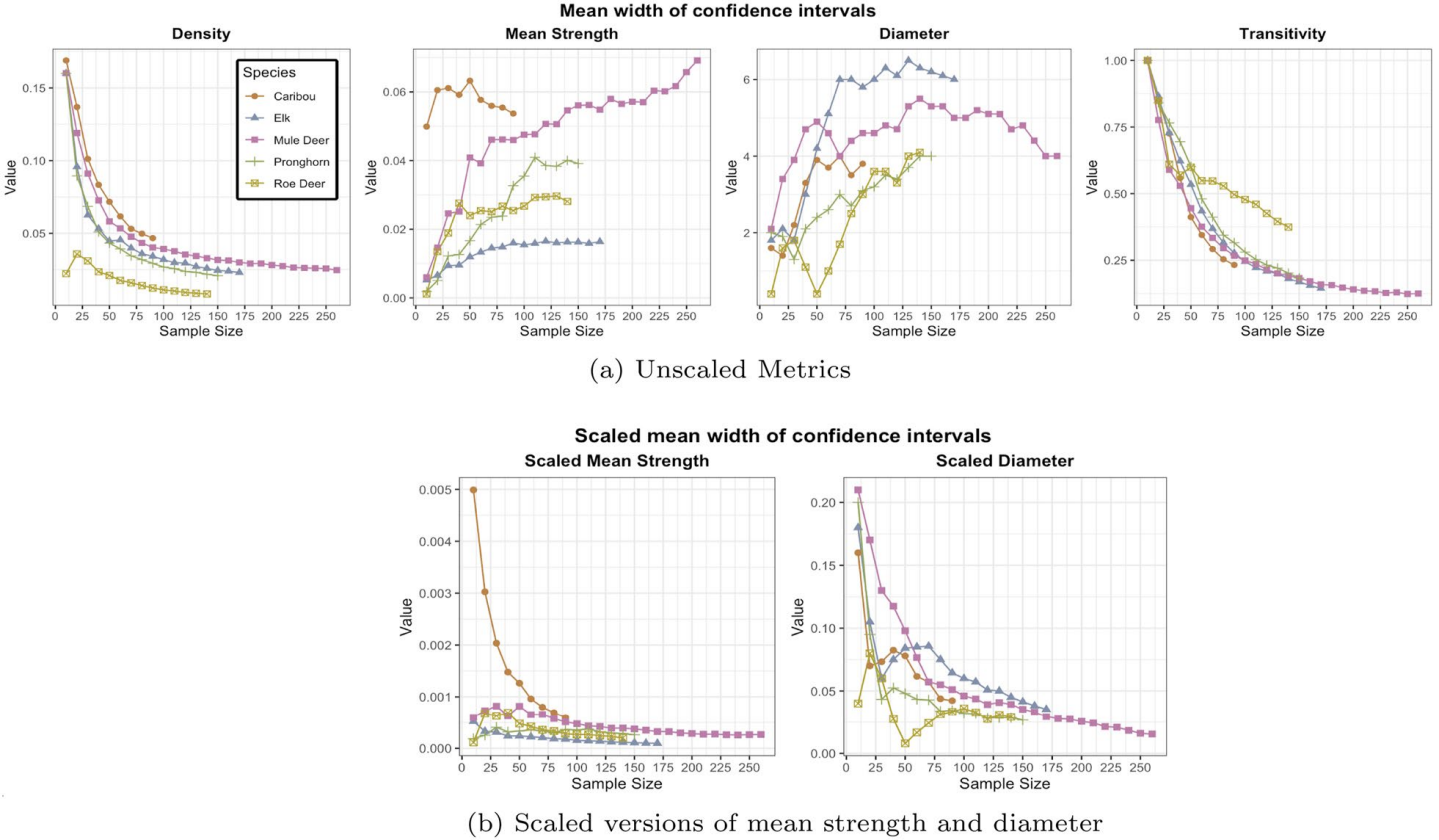
The plots show the mean widths of 95% confidence intervals obtained from bootstrapped sub-samples of a network. The mean widths of density and transitivity increase with lower sample size, which indicates increasing uncertainty around the point estimate of the network metrics. However, the pattern is reversed for mean strength and diameter because the values for these two metrics are directly affected by the number of nodes present in the network. Therefore, we consider scaled versions of these two metrics where the number of nodes at each level scales the values

We used bootstrapping to investigate the extent of uncertainty in the values of network metrics and obtain confidence intervals for each network metric. To observe how uncertainty varies with respect to lowering sample sizes, we plotted the width of confidence intervals against the sample size (Fig. 5a). For the network metric density and transitivity, the mean width of the confidence intervals increased with decrease in sample size for all five species. Mean width for density remained comparatively low for as few as 50 samples but began to increase below that value for all five species. For transitivity, the width remained less than 0.2 when at least 100 individuals were tagged for all species but roe deer. The minimum width for roe deer was 0.4, even when all the individuals in the sample were considered. At smaller sample sizes, mean width approached 1 for all the five species, which is the maximum value transitivity can attain for any network. Therefore, to check that these confidence intervals were not too wide and increased the likelihood of Type 1 errors, we compared two non-overlapping sub-samples from the observed sample to check for significant results (See Assessment of bootstrapping algorithm). This ensured that the bootstrapping algorithm does not generate spurious statistically significant results.

For mean strength and diameter, the width of confidence intervals did not increase with a decrease in the sample size. This is because the values of these metrics were directly affected by the number of nodes in the network e.g., say there were N nodes in the network, the possible degrees of a node could be anywhere between 0 and N-1; however, if we remove M (< N) nodes from the network, the new possible value for the degree would lie between 0 and (N-1)-M, which resulted in a narrower width of the confidence intervals. Therefore, it is better to consider the scaled versions of these metrics where they are scaled by the number of nodes in the network (Fig. 5b). The scaled versions of these metrics followed a similar pattern to transitivity and density. The width of confidence intervals increased with a decrease in the number of individuals sampled. Some fluctuation in this pattern was observed for the density and scaled diameter for some species, when the sample size was low (e.g., roe deer sample). Depending on the selection of nodes in the sub-sample, the value of the diameter in the sub-sampled network can reach extreme values at each level of sub-sampling.

The analysis of confidence interval widths highlighted the extent to which uncertainty increased as the number of tagged animals decreased. Researchers are advised to apply this bootstrapping method to establish confidence intervals around their observed network statistics. Bootstrapping method allows for calibrated estimation of confidence intervals, enabling the users to correctly perform hypothesis testing. For instance, they can correctly determine whether their particular data can be considered to be different from another dataset or if there are seasonal differences, among other things.

#### Step 4. Correlation analysis between node-level metrics of partial and full networks

**Fig. 6:**
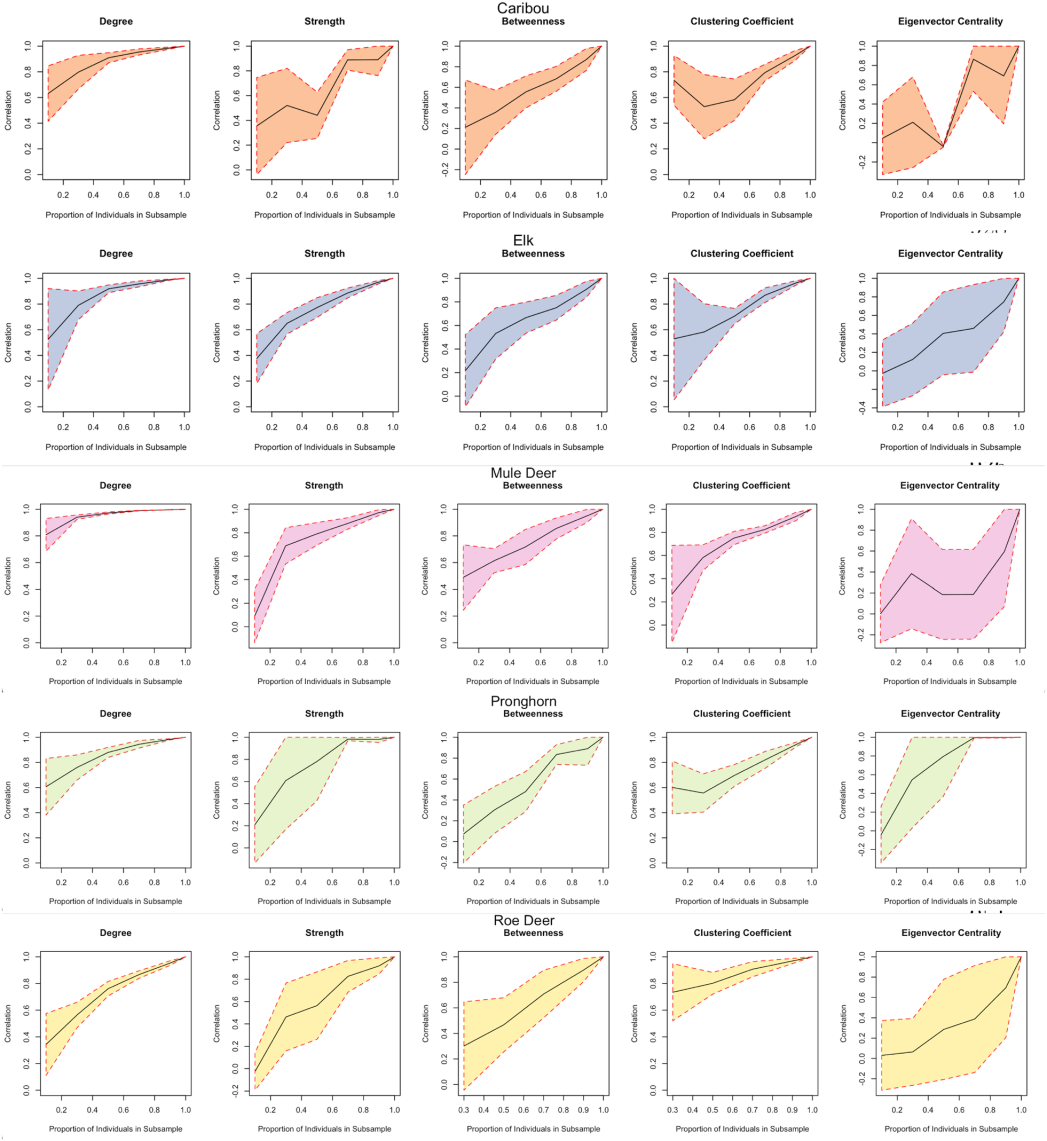
The plots show the correlation of node-level network metrics of the sub-sampled nodes and the same nodes in the observed networks. The black line in the plots indicates the mean correlation coefficient value and the colored region depicts the standard deviation of the correlation values at each sampling level. For example, degree value remains highly correlated with comparatively low standard deviation, even at lower sampling levels

The correlation of all network metrics between the sub-sampled and observed network declined as the proportion of sub-sampled nodes in the network decreased (Fig. 6). However, the pattern and rate of decline were different across network metrics. Degree remained well correlated for mule deer when as few as 10% of nodes were sub-sampled. Mean correlation coefficients of strength, betweenness, and clustering coefficient declined almost linearly with a decrease in the sub-sampling proportion, with slightly more variance in caribou values than mule deer values. For both species, the values for eigenvector centrality became unreliable with high variability even when 90% of the individuals were present in the sub-sample in most of the sub-sampled networks, suggesting that it was a poor measure to use in this case.

#### Step 5. Bootstrapped confidence intervals for node-level network metrics

**Fig. 7:**
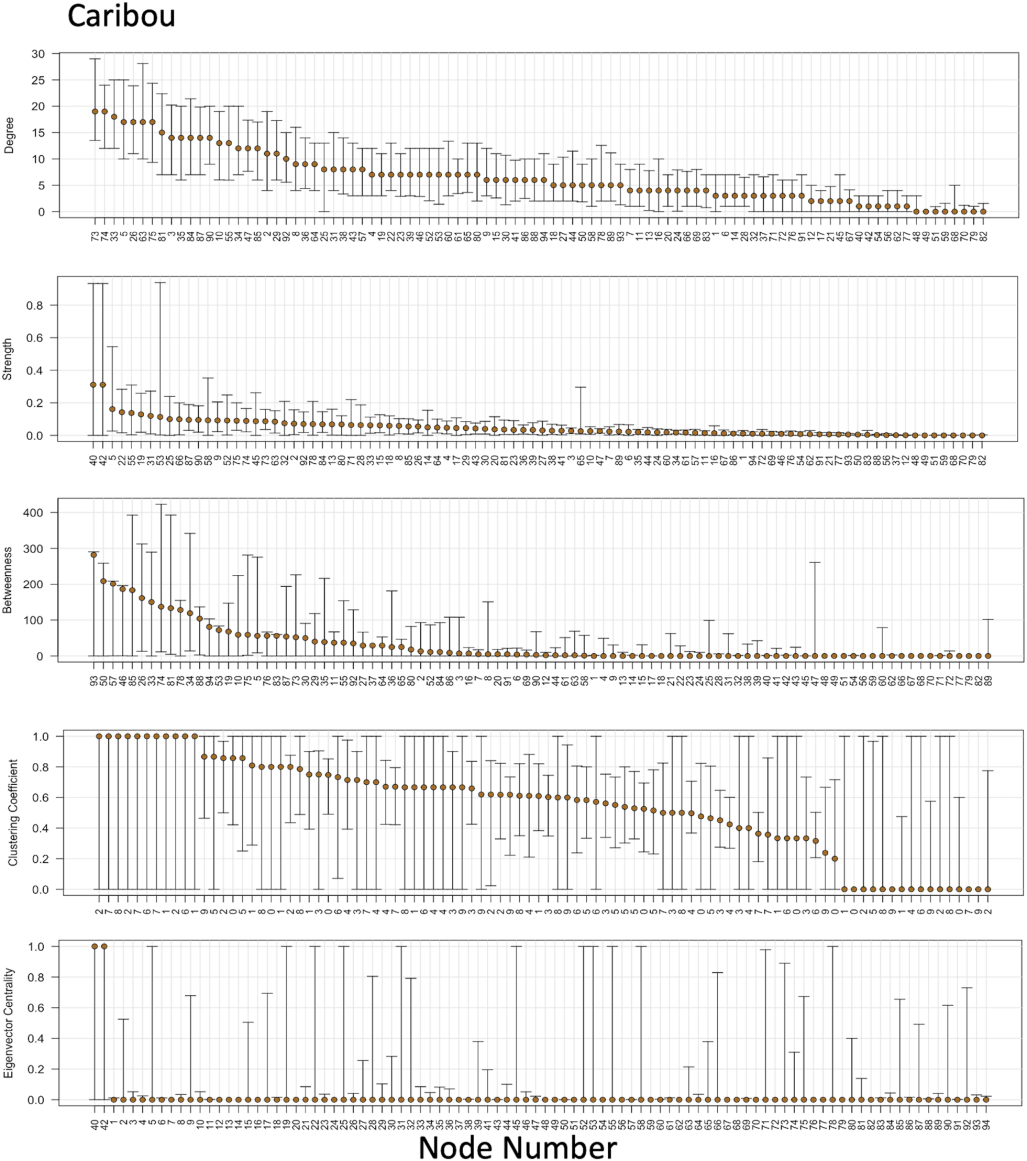
From top to bottom, node-level caribou network metrics (and associated 95% confidence intervals) for degree, strength, betweenness, clustering coefficient and eigenvector centrality, respectively. The nodes are sorted by decreasing order of observed metric to improve readability

We plotted the observed network metric values alongside the confidence intervals for each node of the caribou social network as an example in Fig. 7. The nodes were sorted in decreasing order of the observed metric value to improve readability. Uncertainty varied depending on the selection of the nodes in the sample (Fig. 7). Moreover, the confidence intervals overlapped indicating that the observed rankings could change depending on the selection of individuals in the sample. In addition, some nodes were more likely to have a higher degree than observed, while others were more likely to have a lower degree than observed. The node with the highest strength value has zero in its confidence interval, indicating no significant relationship with its neighbours. Interestingly, its true strength could be over 0.8. The node with the highest betweenness value of over 250 in the observed sample may have a value as low as 10 in some other sample. The observed values, in this case, were not just influenced by immediate neighbours but also by the choice of other nodes in the sample. A high uncertainty in clustering coefficient values was influenced by not just the number of neighbours sampled but also the number of times they were sampled. The network metric eigenvector centrality showed polarisation in node values. Twelve nodes with observed values near zero showed a tendency to reach as high as 1, which could be identified using the bootstrapping technique. These results for the uncertainty in node-level network measures also explain the magnitude of bias and uncertainty in global network metrics that we noticed while subsampling. Because density is a summary of degree, low uncertainty in the node-level measurements of degree and a linear trend in the ranks explain the low uncertainty and bias in the global network metric of density. The plots for eigenvector centrality, on the other hand, do not have a linear trend in ranks with a few nodes having a high observed value with a considerable uncertainty. The presence of these nodes in the subsample significantly impacts the average eigenvector centrality of the network, resulting in high variance in correlation coefficient values.

**Box 1:** Line-up of steps

1.	Define the network edges by choosing a sensible distance threshold based on the research question, species sociality, and information obtained from the data (see section Identifying Associations and Network Construction for more details).
2.	Check if the interactions captured by the sample are non-random with the help of network permutations. Network metrics can be deemed suitable after assessing whether they capture non-random associations via network permutations (Step 1).
3.	Identify stable network metrics concerning the species and the available sample using sub-sampling from the observed data (Step 2).
4.	Identify the minimum sampling effort required to determine the network properties that are different from a randomly generated network by comparing the sub-sampled networks from permuted data sets with the sub-samples of the observed data (Step 2).
5.	Obtain confidence intervals around the point estimates of network metrics using the bootstrapping algorithm, which also takes into account the autocorrelated structure of telemetry relocation data. The width of confidence intervals can also be analysed for lowering sample sizes (Step 3).
6.	To assess which node-level network metric remains least affected with lowering sample sizes, obtain a correlation coefficient between the node-level metrics from the observed sample and the same nodes from the sub-sample. The local network metrics with a high correlation (>0.7) are expected to be more stable and should be chosen for further analysis as they are more likely to represent the position of individuals in the network, similar to their position in the full population (Step 4).
7.	To estimate the uncertainty in the observed values of node-level network metrics, obtain confidence intervals around the point estimates for each node (Step 5).

Results for the workflow analysis of the fully observed population of Phoenix Park are presented in Analysis of fallow deer population in Phoenix Park in detail. The patterns in those results are strikingly similar to what we observed for the five ungulate populations tracked by means of satellite telemetry; however, a high correlation and low uncertainty was observed for both node-level and global network metrics at even lower levels of subsampling, owing to the high proportion of individuals observed in the population.

## Discussion

We have presented a five-step workflow to assess the stability of global and local network metrics and obtained uncertainty measures around the point estimates of the network metrics obtained from GPS telemetry observations. This workflow can be used to obtain reliable inferences about the structure of a social network and we have demonstrated it on telemetry observations of five species of ungulates. First, using data permutations, we found whether the sample data captured non-random aspects in all or some of the network metrics: this was entirely true in roe deer, for instance, whereas in other species such as elk the data collected were able to capture non-random associations only with edge density and mean strength. Two of the five species data provided evidence that all aspects of the network differed from random, while the other three showed only specific aspects deviating significantly. This highlighted a key strength of our five-step protocol. By systematically evaluating different network metrics, the protocol helped determine if the collected data was suitable for addressing specific hypotheses. This is a key step in our approach, because at this stage the researcher can make the decisions on whether to use such metrics in their study case. Second, sub-sampling from the observed sample revealed density as the most unbiased measure of animal networks with low uncertainty, even at small sub-sampling proportions. Third, we introduced bootstrapping techniques for animal social networks, which allowed us to compute confidence intervals around the point estimates of the network measures. Density and scaled version of mean strength emerged as two of the most robust network metrics. Fourth, correlation analysis between the node-level metrics of the observed network and the sub-sampled network highlighted the network metric degree to be most correlated with the observed network metric values, even at 40% of sub-sampling levels. This means that if the information about sampling proportion were available, the relative degree of each individual could be used to estimate the true degree distribution. Lastly, we were able to establish confidence intervals for each node’s network measure by performing node-level bootstrap analysis. This not only identified the nodes of the social network with extreme metric values for a given network, but also the nodes that have a tendency to attain extreme values not observed otherwise, owing to the selection of other nodes in the sample. Furthermore, for any specific node of interest, we could determine if it was more likely to have a greater true value than observed or a lower true value based on the length of its upper and lower confidence intervals. All of the workflow steps are provided as functions in the R package, aniSNA [45, 46] allowing users to undertake such an analysis of their data. We have summarised the steps that should be taken to perform this analysis in Box 1. Line-up of steps.

Despite being a commonly used tool to understand animal ecology [42, 49, 64, 68, 75, 76], social network analysis can be challenging when applied to real-life datasets [15, 26]. [15] performed tests to demonstrate a distinction between networks built using different interaction and proximity techniques. Similar tests performed by [26] illustrated that the conclusions by [15] cannot be generalized across species. A researcher’s choices during the data collection and the analytical stage affect the networks produced. Therefore, the inferences generated may not reflect true characteristics and can be highly sensitive to these decisions [15, 32]. Furthermore, the information available about the sampling protocols may be incomplete. However, this does not imply that social network analysis should not be conducted on such data. While understanding the limitations of the data one is working with, it is prime to use statistical methods that would help extract as much information as possible, along with details about the uncertainties due to partial data and sampling strategies. Performing permutations to randomise autocorrelated GPS data stream [27, 77] is a first step to check if the selected network metrics capture the non-random aspects of social interactions. Different network metrics capture different aspects of the network; some networks may have more non-random elements than others, depending on the species’ sociality and the sampling strategies adopted to collect the data. Our analyses have helped highlight the network metrics that distinctively capture these non-random aspects. Researchers are advised to not use this permutation approach for “searching” the non-random metrics. Instead, they should use it to evaluate the adequacy of their dataset and determine if the network metrics, selected based on expert ecological knowledge, effectively capture non-random aspects. Also, the null models obtained here cannot fully disentangle social preferences from fine-scale spatio-temporal behaviour patterns as those are not attainable with association data alone. Based on our analyses of the five species, the network metric mean strength is recommended to be used as an assessment metric to identify if the captured interactions are significant enough to generate reliable analysis results. Indeed, apart from the four network metrics we chose to work with, it could be helpful to run this analysis on other network metrics that seem suitable for the particular research question (e.g., coefficient of the variation of edge weights [57]). Once a network metric is chosen by the user, further analysis of the available dataset can be carried out to answer the research question. Researchers should keep in mind that this approach is particularly suited (but not limited) to autocorrelated telemetry relocation, although its use could be expanded to more rarefied observational data (e.g., low frequency observations of individually recognizable individuals in a population, similarly to what we have done with the fallow deer study case).

Caution should be taken while reporting the values of social network metrics when the sample size is small relative to the population [29, 54]. As a general rule, the smaller the sample size, the more considerable uncertainty can be expected in the observed values. However, some network metrics remain unbiased despite significant uncertainty, whereas others would become biased as the sample size decreases. Sub-sampling from the observed sample and permuted versions of the network revealed helpful information regarding the stability of certain metrics and the proportion of individuals required to ensure a non-null network. For the samples we used, density and transitivity emerged as the more stable metrics, remaining unbiased when as low as 10% and 30% of the individuals were sub-sampled, respectively. Network metrics such as mean strength became biased as we lowered the number of nodes in the sub-network. However, it was well characterised by a linear relationship. The choice of individuals in the sub-sample greatly affected some network metrics such as diameter. However, it always tended to plateau when the proportion of sampled individuals increased. Sub-sampling from permuted versions of the data and comparing it with the distribution of sub-samples from the observed network revealed the minimum sub-sampling level required to ensure a non-null network structure. [22] investigated the effect of sampling effort on the accuracy of social network analysis and concluded that increased sampling intensity may not always increase the accuracy of network measures especially when the sampling regime was already very intense.

Reporting point estimates for metrics is not enough especially when a large proportion of individuals in the population is not monitored. It becomes equally important to communicate uncertainty around those estimates [54, 80]. We presented bootstrapping as a powerful approach to evaluate confidence intervals around the point estimates of global and node-level network metrics from the observed data. Bootstrapping enabled us to assess the extent of variation in the global network metrics if a different set of individuals was sampled from the population. The network metrics of density and scaled mean strength had low uncertainties, even at small sample sizes for the data of five species that we used. Obtaining bootstrapped confidence intervals for node-level metrics allowed the characterization of social network values at individual level along with their uncertainty values which can be used in further analyses and hypothesis testing when the researchers also have other information on the single individuals (e.g., movement rate, behaviour, life history traits such as mating success and survival).

Past studies have tried to uncover how the values of node-level metrics are affected when the sample sizes are lowered [17, 34, 67]. We have built on the simulation studies of [67] by performing the correlation and regression analyses between the local metric values of the nodes in the entire sample and the values of the same nodes in a smaller sub-sample of our empirical data. With correlation analysis at various subsampling levels, we determined how the correlation rate decreases as the sub-sample size is lowered. Out of the five node-level metrics that we tested, degree performed better, with high correlation even at low sample proportions. Also, we recommend not using eigenvector centrality if a large proportion of the population sample is not available as the metric being a higher-order statistic lacks robustness and, therefore, is highly sensitive to the selected nodes. This was confirmed through our node-level analysis on the five datasets and also agrees with the simulation study by [67]. We conclude that care should be taken while comparing the metric values between nodes when a small proportion is tagged from the population. Indeed, the social network positions captured by a small sample may not reflect the actual positions in the network in such cases.

The primary goal of the proposed workflow is to quantify the bias and uncertainty in empirical data, and the analysis of Phoenix Park fallow deer population has effectively verified the purpose of this workflow. The analysis showed how uncertainty and bias in both global and node-level metrics vary with decreasing sample proportions. Including Phoenix Park data has demonstrated the robustness of our workflow, something not achievable with simulated data. Simulated data lacks the complexity of empirical data and sets unrealistic standards for measuring uncertainty in animal social networks.

**Fig. 8:**
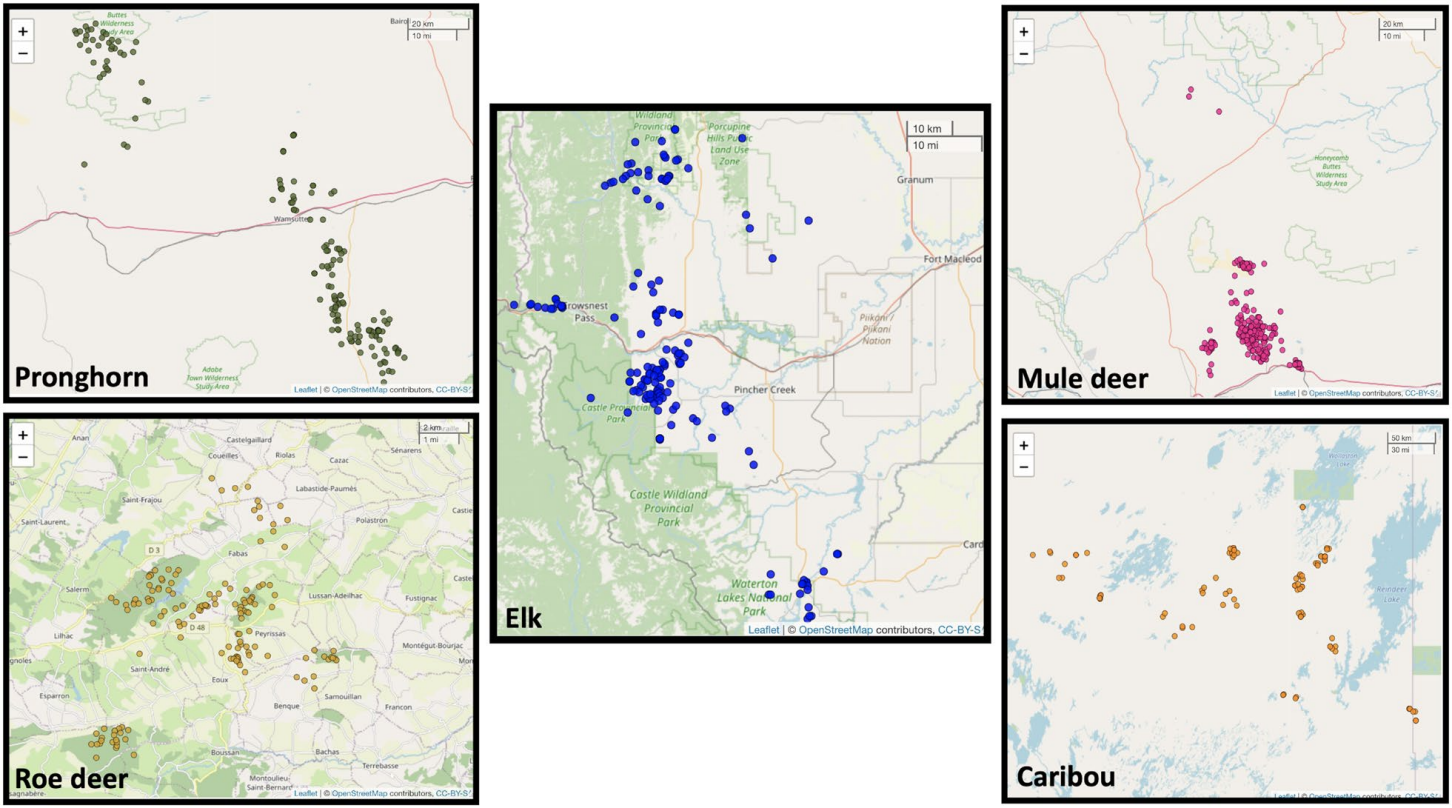
Plot of the initial locations for the individuals belonging to five species in the study. The scale bars are present at the top right corner of each map. The distribution of these locations explains some of the differences in the values of network metrics. For example, the initial capture locations of mule deer are spatially very close, which is also reflected in the network metric values of the final network of associations. As a result, the mule deer network has the highest mean strength and mean degree (Table 3). In contrast, the roe deer network has the lowest mean strength and mean degree, partially explained by their six spatially separated capture sites

The goal of this paper was not to make inferences about the network characteristics of an entire population of any species but to present ways that assist in analysing how different network metrics scale under downsampling depending on the availability of data. As a matter of fact, despite having access to large telemetry samples from five different species, they represent a subset of a population with unknown size. The methods discussed here can help pinpoint useful social network metrics that remain robust when trying to answer a particular research question. Those metrics that suffer from data thinning and become unstable should not be used with telemetry data, which is typically used to monitor a small proportion of the actual population. Also, we used data from multiple species of large herbivores with very different ecology and characteristics, including migratory/non-migratory from very social to solitary species. Despite our a priori disregard of the ecology of the five species for the reasons stated above, we found interesting differences among them which deserve to be discussed here. Firstly, the fact that the data collected from a more solitary species such as roe deer (See Table 3) better capture the non-randomness of the association compared to more gregarious species such as elk suggests that sample size (in proportion to the actual population size) should be higher in more gregarious species especially while using global network metrics for analysis. In addition, sampling regimes can affect the social network patterns and related ecological inference. For example, a high-density value of the roe deer network as compared to the distribution of null networks could be due to the fact that sampling was conducted across six spatially separated capture sites (within 10 x 10 km). This results in very low density values when the data is permuted across these six clusters (Fig. 8). Instead, the mule deer’s initial locations (Fig. 8) show that the network is already very dense. In the permuted versions of the raw data, the number of random interactions is similar to the number of observed interactions, resulting in an observed network density value similar to the density of permuted versions of data. In other words, the sampling strategy (location of the capture sites) may affect the spread of individuals and the density of the respective network structures, therefore researchers need to focus on the ecological interpretation of their social network results after having taken into account the possible bias introduced by sampling strategies. The five-step protocol provides useful tools for assessing uncertainty and can deliver reliable results when used with representative samples. However, it cannot address or rectify inherent biases in the initial sampling. Researchers need to be aware of this limitation and recognize that it is not intended to correct any pre-existing sampling biases in the data.

Numerous papers have examined the conceptual properties of centrality measures to assist animal social network researchers in selecting the most meaningful and valid measure for their research question and the available data [9, 29, 35, 36]. The performance of network centrality measures under various sampling regimes and the species sociality could vary to a great extent [17, 23, 38] and our work confirms this. Future work involves analysing the effects of observation frequency and duration on the accuracy of network metrics. For example, it could help to understand if it is better to observe individuals for a longer duration with low temporal resolution or a shorter duration with high temporal resolution. In our analysis, sub-sampling on the observed samples was random. However, this differs from the sampling strategies adopted in real life. [71] investigate the effects on estimates of key network statistics when central nodes are more/less likely to be missing. Application of our methods to determine how the network metrics scale when a different sampling strategy is adopted would be valuable (e.g., whether it is better to sample entire groups, or focus on greater sampling frequency of individuals). Another vital direction forward is to assess the methods presented in this paper to be tested on the GPS telemetry data of the entire population. If data on the whole population is available (e.g., a fenced one), it will be interesting to perform these methods on a subset of that and test if the predictions align with the true values. This approach could facilitate more robust measures to evaluate the reliability of social network metrics derived from incomplete data. In future research, there is also a potential to utilize the protocol to identify bias through deliberate subsampling with known biases. This deliberate manipulation would enable a deeper exploration into the detection of biases and their effects on network metrics. This approach would offer insights into the impact of bias on metrics, aiding in comprehension and potentially informing the development of corrective methods.

## Conclusions

Along with all of the advantages in understanding animal ecology, SNA presents certain challenges that hinder ecologists from using it to its full extent. We addressed a few of those challenges in this paper and introduced a five-step workflow to assess the suitability of available data for SNA and extract information for further analysis. The methods are also provided as easy-to-use functions in an R package aniSNA [45]. This package allows ecologists to directly apply these statistical techniques and obtain easily interpretable plots to provide statistical evidence for choosing a particular network metric or the choice of individuals tagged for the study [46]. The fact that researchers can compute confidence intervals around their point estimates unleashes new research opportunities, such as tackling specific hypotheses. For instance, researchers can estimate network metrics in a sample population when it is disturbed by human presence to be compared to when it is not disturbed, and the ability to assess the overlap of respective 95% confidence intervals would allow making inference on the effect of human disturbance on sociality. Likewise, this approach can be used to compare social networks within and across populations as a function of temperatures, presence of predators, or different wildlife management strategies, unleashing a range of ecological questions using SNA and related statistical tools.

## Figures and Tables

**Fig. 9:**
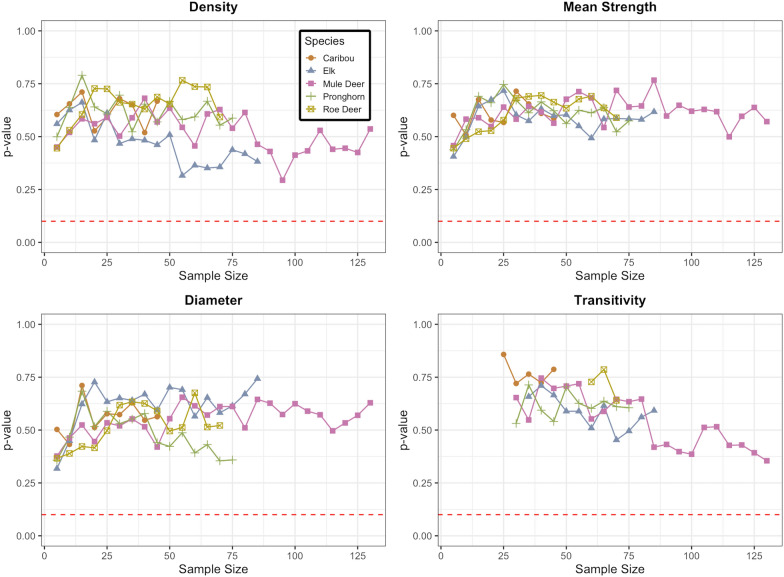
Mean of p values for the difference in network metrics values for two non overlapping sub-samples of the observed sample. The two non-overlapping sub-samples are bootstrapped 1000 times and *p*-values are computed for to check for the significance of difference. This process is replicated 10 times to obtain mean of p values. The red dotted line in each plot corresponds to value 0.1 on the y-axis. All the mean *p*-values lie well above this dotted line

**Fig. 10:**
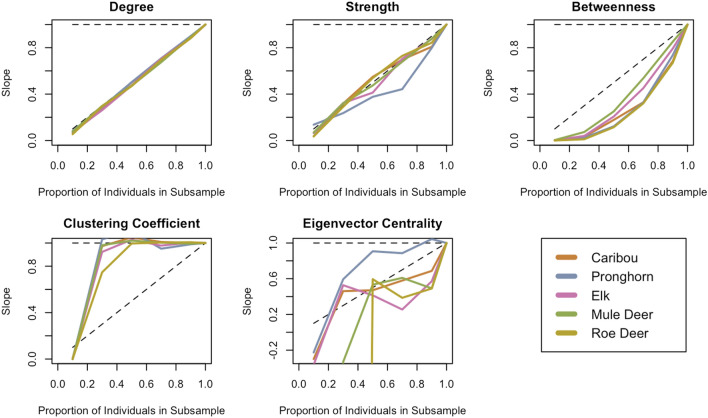
Regression analysis of local network metrics between the nodes of partial and observed network. In each plot, X-axis denotes the proportion of nodes in the sub-sample and Y-axis shows the corresponding value of the slope of regression calculated by regressing the local network metrics of sub-sampled nodes and observed network nodes

**Fig. 11:**
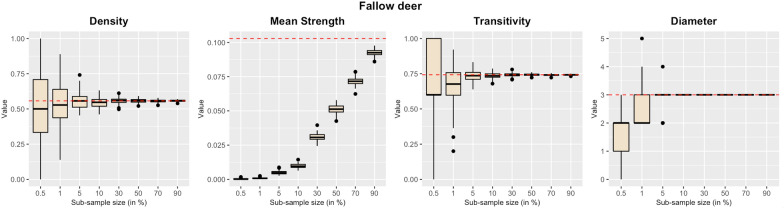
Effect of sub-sampling on four global network metrics. The horizontal red line in each plot represents the metric value in the observed network. The boxplots denote the distribution of network metric values obtained from the observed networks by taking 100 sub-samples at each sampling level ranging from 0.5 to 90% of the observed network. Note that - to improve plot readability - the x-axis categorically represents the level of sub-sampling and the gap between each level is not true to the scale

**Fig. 12:**
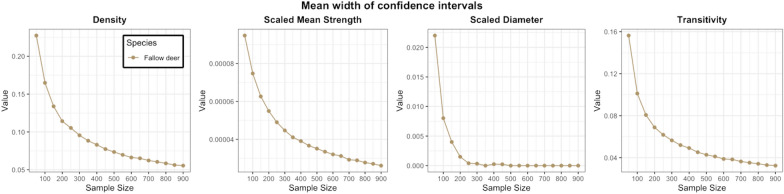
The plots show the mean widths of 95% confidence intervals obtained from bootstrapped sub-samples of a network for the four global network metrics

**Fig. 13:**
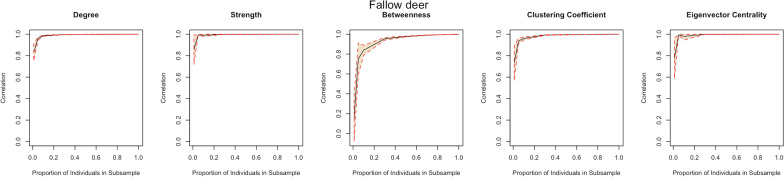
The plots show the correlation of node-level network metrics of the sub-sampled nodes and the same nodes in the observed networks. The black line in the plots indicates the mean correlation coefficient value and the colored region depicts the standard deviation of the correlation values at each sampling level

**Fig. 14:**
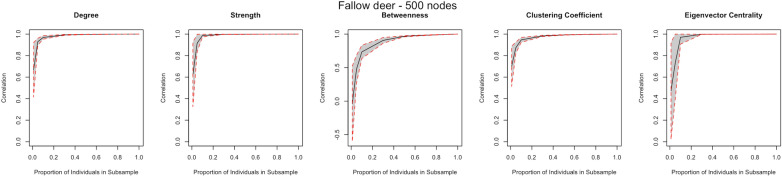
The plots show the correlation of node-level network metrics of the sub-sampled nodes and the same nodes in the network consisting of 500 nodes chosen randomly from the total number of nodes (n = 951). The black line in the plots indicates the mean correlation coefficient value and the colored region depicts the standard deviation of the correlation values at each sampling level

**Fig. 15:**
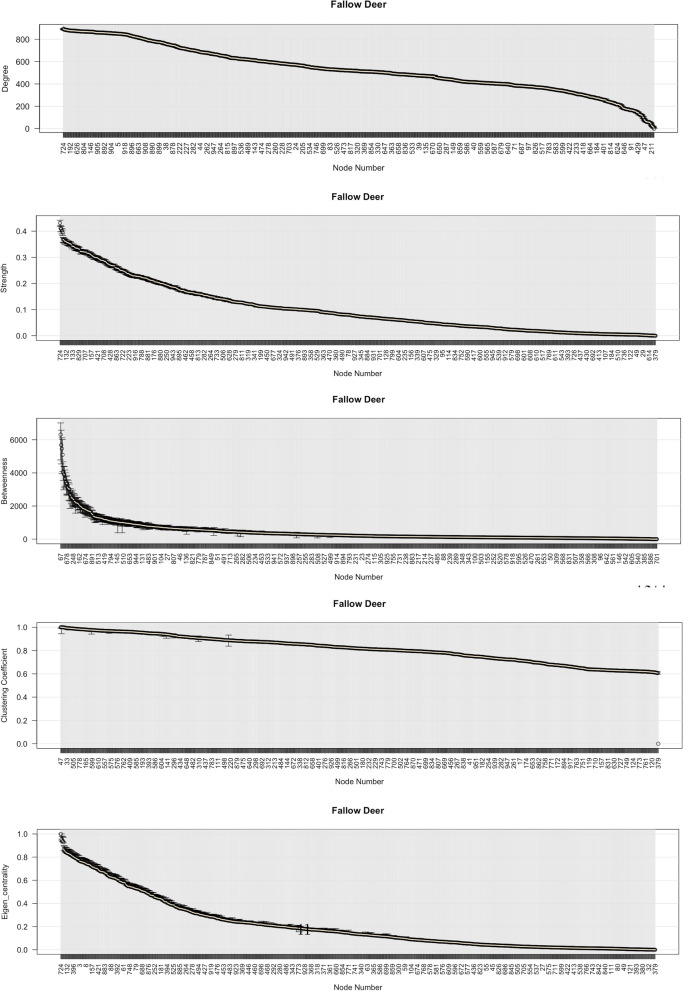
From top to bottom, node-level fallow deer network metrics (and associated 95% confidence intervals) for degree, strength, betweenness, clustering coefficient and eigenvector centrality, respectively. The nodes are sorted by decreasing order of observed metric to improve readability. The widths of confidence intervals are narrow for all nodes and metrics except for a few nodes having high betweenness values

**Fig. 16:**
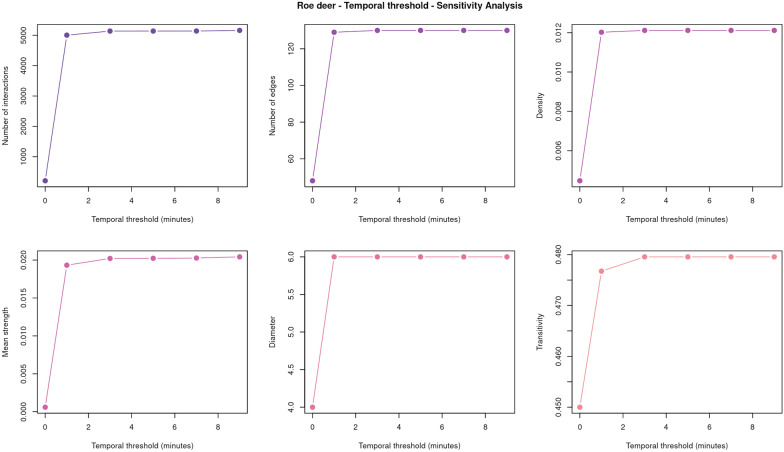
From top to the bottom, node-level elk network metrics (and associated 95% CI) for degree, strength, betweenness, clustering coefficient and eigenvector centrality, respectively. The nodes are sorted by decreasing order of observed metric to improve readability

**Fig. 17:**
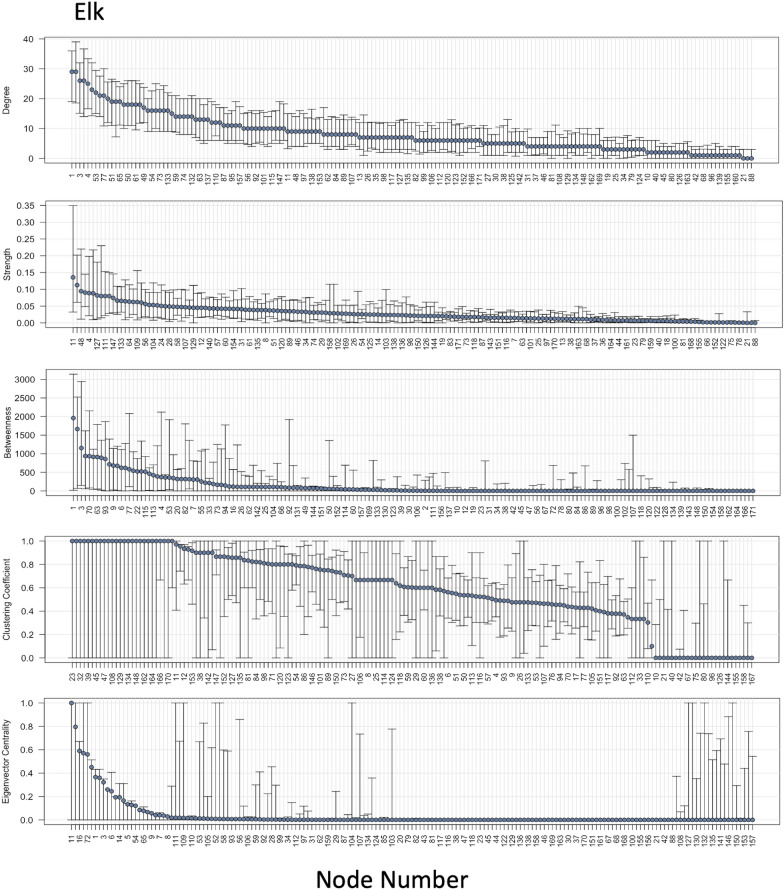
From top to the bottom, node-level mule deer network metrics (and associated 95% CI) for degree, strength, betweenness, clustering coefficient and eigenvector centrality, respectively. The nodes are sorted by decreasing order of observed metric to improve readability

**Fig. 18:**
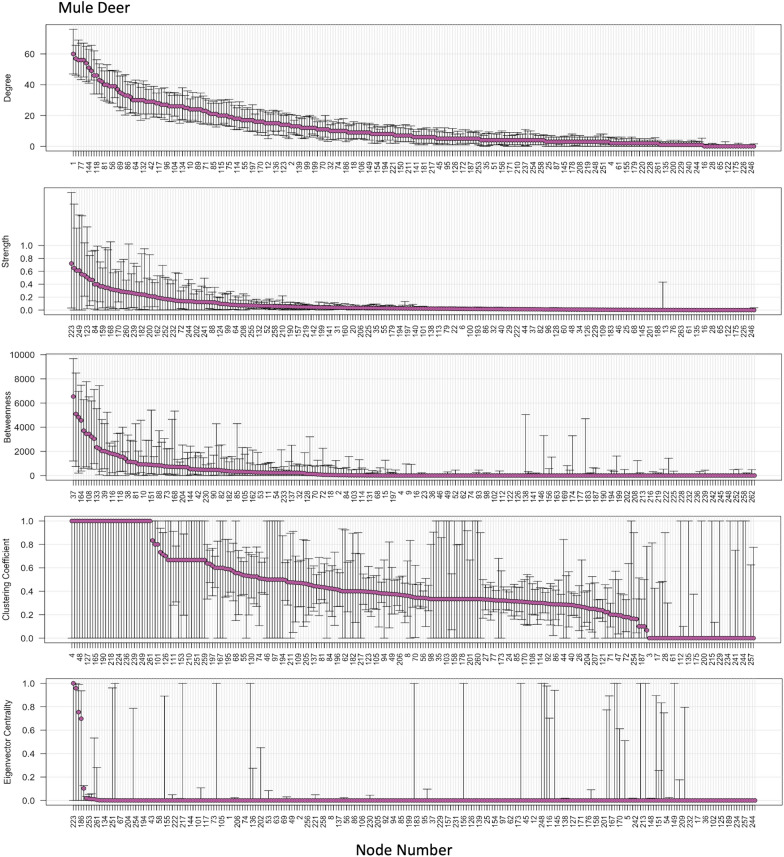
From top to the bottom, node-level pronghorn network metrics (and associated 95% CI) for degree, strength, betweenness, clustering coefficient and eigenvector centrality, respectively. The nodes are sorted by decreasing order of observed metric to improve readability

**Fig. 19:**
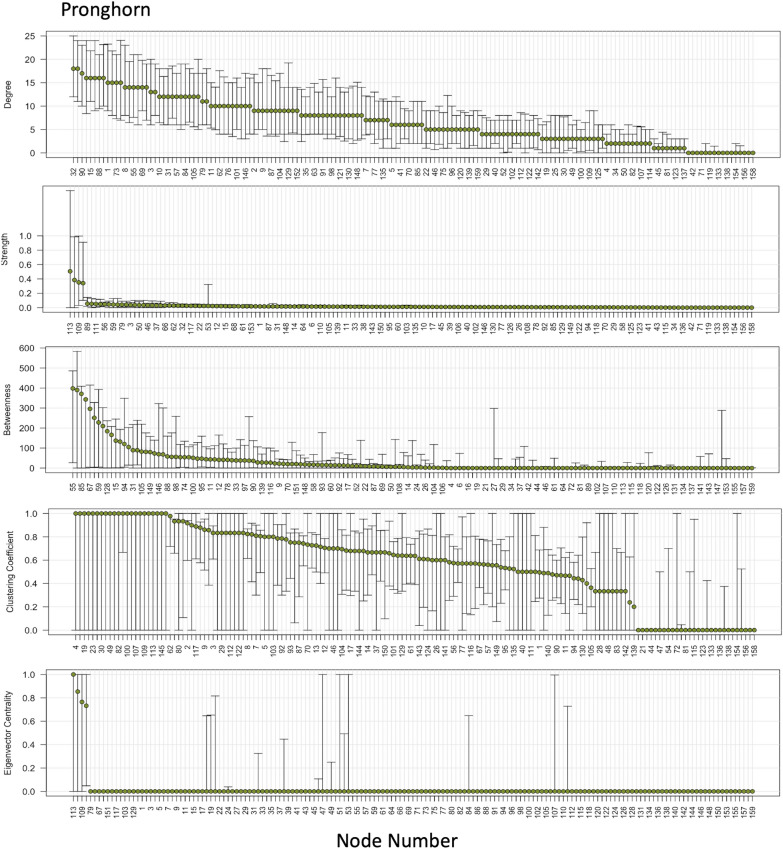
From top to the bottom, node-level roe deer network metrics (and associated 95% CI) for degree, strength, betweenness, clustering coefficient and eigenvector centrality, respectively. The nodes are sorted by decreasing order of observed metric to improve readability

**Fig. 20:**
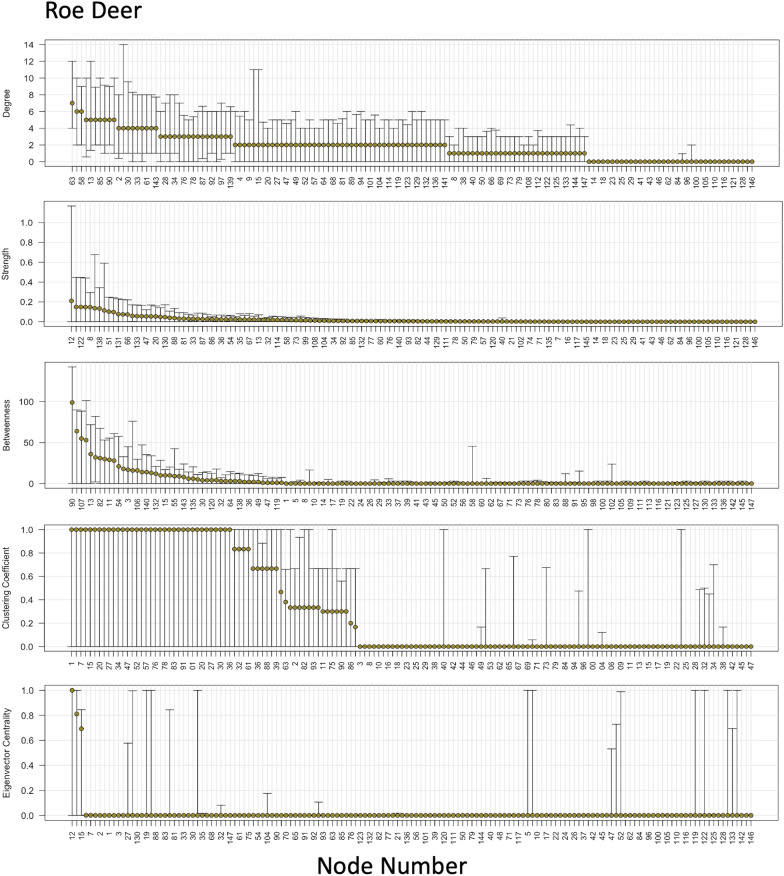
Sensitivity analysis of the network metric values with respect to different values of temporal threshold. The x-axis in each plot represents the value of temporal threshold in minutes and the y-axis corresponds to the value of global network metric for the network obtained by choosing a specific temporal threshold value

## Data Availability

The data that support the findings of this study have been made publicly available in a GitHub repository at https://github.com/PrabhleenKaur19/SNA_data.git. Please note that the geographical locations in the dataset have been jittered to protect privacy.

## Appendix 1: Assessment of bootstrapping algorithm

We bootstrapped two different subsamples of a social network at each subsampling percentage. Given that the subsamples were taken from the same population, any significant differences should be attributed to overly confident bootstrapped intervals and non-significant results were to be expected. We repeatedly sampled pairs of networks at each subsampling level and computed the *p*-value for a significant difference between the two network metrics, as determined using a two-tailed t-test as per [74] (see Step 3. Bootstrapped confidence intervals for global network metrics for more details). If the subsampled networks do indeed provided noisy estimates of the network metric calculated across the population and the bootstrap algorithm did not return intervals that were too narrow, the B bootstrapped *p*-values would not contain more than approximately $\alpha$B values less than $\alpha$ (for any significance level $\alpha$). Indeed, the *p*-values would ideally be uniformly distributed between 0 and 1. We do not perform tests of our bootstrapping approach with regard to power / Type 2 errors because, as with all hypothesis tests, the power to correctly identify real differences in network metrics between two different social networks depends not only on sample size but also on the magnitude of the differences. The larger the sample size and the larger the true difference, the greater the power to identify that the networks differ.

For all the network metrics, *p*-values for difference between two samples is non-significant for all the five species (Fig. 9) which indicates that our bootstrapping approach is not overly sensitive i.e. return too many false positive results when used to compare the network metrics of two networks.

## Appendix 2: Regression analysis between node-level metrics of sub-sampled and observed networks

While analyzing node-level metrics from a partially sampled network, it is useful to know the extent by which the position of an individual in the population controls its position in the sampled network and how this control depends on the choice of individuals in the sample or density of the population.

To answer this, we perform regression analysis such that the values of node-level metrics in the sub-sampled networks are regressed on the values of those nodes in the observed network. As in the correlation analysis, the individuals are further sub-sampled to analyse the effect. Sub-sampling is done at 10%, 30%, 50%, 70% and 90% levels and is repeated 10 times at each level. The mean slope of regression is calculated for the five network metrics of degree, strength, betweenness, clustering coefficient and eigenvector centrality at each level for each run of the simulation. The slope of regression describes how the value of network metrics calculated in each partial network relates to its value in real network as per [67].

The slope of regression is plotted against sampling proportion (Fig. 10) The accuracy of node-level metrics from partial networks is highly dependent on the metric being used. In agreement with the results of the simulation study by [67], the accuracy of local measures of degree and strength decreases linearly in direct proportion to the proportion of individuals subsampled. In contrast, the accuracy of eigenvector centrality does not depend on that proportion in any of the networks except for elk network. The value of the slope of regression for betweenness decreased non-linearly for four of the species that have low number of individuals tagged and followed a near-linear pattern for mule deer that has high number of individuals observed. The accuracy of the clustering coefficient did not decrease till the level of identified individuals was as low as 50% but declined rapidly at different levels after that for each of the species.

[67] suggests that the strong relationship between the proportion of individuals sampled and the accuracy with which local measures (degree and strength) predict the actual value of an individual’s centrality is notable. This implies that it is possible to correct for this effect if the proportion of sampled individuals in a population is known.

## Appendix 3: Analysis of fallow deer population in Phoenix Park

The data for the five species of ungulates reported in the main manuscript and chosen for the analysis and demonstration of the five step workflow consisted of a subset of the populations for which the exact sizes were unknown, therefore the precise proportion of individuals sampled within each population was unknown. However, to demonstrate the validity of our workflow, we had included a sixth dataset in this analysis as supplementary material belonging to the fallow deer *(Dama dama)* population of the Phoenix Park in Dublin, Ireland. This dataset was different from the five datasets included in the main manuscript for the following reasons. Firstly, fallow deer in this population are routinely captured and ear-tagged within a few days after birth [2], and observed by intensive monitoring programs [39], meaning that precise and accurate estimates on population size as well as on the proportion of ear-tagged individuals are available for this population. Each year, with over 500 out of 600 individuals tagged, this dataset offers a highly robust social network analysis and serves as an ideal choice to validate the five step protocol. This is because in similar studies, GPS telemetry usually covers less than 10% of the total population.

Secondly, fallow deer in Phoenix Park are monitored via standardised visual observations [3, 39] usually at a frequency of a few observations per week opposed to the high frequency data collected via satellite telemetry, therefore giving us the opportunity to verify the effectiveness of our protocol on a different type of data than those presented in the main manuscript. Assessing the validity of our approach in a completely different ecological context (peri-urban) and sampling design (visual observations opposed to satellite telemetry) makes our protocol accessible to the broader users and not only limited to satellite telemetry data.

### Study site

The data in this analysis have been collected in the population of fallow deer residing in Phoenix Park, a 709 ha peri-urban park located less than 2 km from Dublin city centre, Ireland (53$^{\circ }$ 22’ N, 6$^{\circ }$ 21’ W). Phoenix Park is one of the largest enclosed urban parks in Europe and hosts a fallow deer population of slightly more than 600 free-ranging individuals (end of summer estimates, including newborn fawns), which are kept to a steady population size by 2–3 yearly culls. The adult population is sexually segregated for most of the year with adult females and males only coming together for mating during the rut season (September to early November).

### Data collection

The data used for this analysis were observational data collected between May 2018 and January 2023. The data included one weekly data collection conducted between August and April each year, whereas the months between May and July were surveyed more frequently (almost every day) because of a concurrent human-deer interaction study (see [39]. The observations were carried out strictly following a stratified sampling design based on time of day, day of the week and area of the park. Whenever a fallow deer group was encountered, the following data were recorded: herd size, identity of all individuals recognizable by unique colour-coded ear-tags, exact proportion of tagged vs untagged individuals, location (using a Garmin GPS hand-held unit) and time of observation. Any herd was defined as a group of 1 or more individuals being within 50 m and in view of each other. For more information on the sampling protocol see [39]—which was defined for the core data collections of May–July, and strictly followed by the weekly surveys from August to April. During the study period,  85% of the overall population was individually recognizable by unique ear-tags.

### Data analysis

From May 2018 to January 2023, nearly the whole population was monitored for this observational data. There were 951 nodes in the resulting network, and over 250,000 interactions were observed. This was a dense network whose summary is given in Table 4.

When analysing the observational fallow deer data, we had omitted the first stage of our five-step workflow. The first step is better suited to GPS telemetry observations where an arbitrary distance threshold is selected to define when two individuals are deemed to be together, therefore null networks are generated by permuting a pre-network data stream to determine if the network structure obtained from the available sample of GPS observations captures any non-random aspects of the association. When dealing with the observational fallow deer data and already defined groups, we therefore moved on with the second step by computing the network metrics that we were interested in.

**Table 4:** Summary statistics for fallow deer data

Species name	Order (nodes)	Size (edges)	Mean degree	Mean strength	Mean betweenness	Mean eigenvector centrality	Mean local clustering coefficient	Density	Transitivity	Diameter
Fallow Deer	951	251,661	529.3	0.103	437.8	0.211	0.812	0.557	0.742	3

#### Analysing subsamples of the observed network (step 2 of the protocol)

We obtained 100 subsamples with the node counts ranging from 0.5 to 90% of the observed network and generated plots similar to those from the sub-sampling analysis of the five species presented in the main manuscript. The results plotted in Fig. 11 demonstrate that even at lower sampling levels of 5–10% of the entire population, density and transitivity are accurately estimated with no bias and small variance. Up to 5% of the monitored population, diameter estimates are highly uncertain, but above that point, they are highly accurate and precise. The network metric mean strength increases with sample size, allowing estimation of population mean strength using linear extrapolation to 100% sample size if the actual population size is known.

#### Bootstrapped confidence intervals for global network metrics (step 3 of the protocol)

We obtained 1000 bootstrapped samples at different levels of sampling with the number of nodes varying from 50 to 900. We calculated the four global network metrics density, scaled mean strength, scaled diameter and transitivity for each bootstrapped network at each level and obtained corresponding confidence intervals. Plotting the width of confidence intervals against the sample size showed a similar pattern to those of the five species we reported in the main manuscript. As expected, with a decrease in the size, the confidence intervals widened. However, it was interesting to note that the confidence interval width for density, scaled mean strength and transitivity remained comparatively low at a 40% sampling level. For scaled diameter, the confidence intervals were narrow even when just 250 out of 900 nodes were sampled. For all metrics, 100 nodes corresponded to markedly high confidence interval width, making it a weak sample to obtain inference for scaled diameter. This analysis, more in general, provides more insight into the sample size required for a given level of accuracy for the social network metric estimate (Fig. 12).

#### Correlation analysis (step 4 of the protocol)

Correlation analysis on the fallow deer dataset representing the vast majority of the Phoenix Park population allowed us to verify whether using this analysis with a significantly smaller subsample of a population (e.g. those reported in the main manuscript) was useful or not. We calculated the correlation between the node-level network metrics of each node in the fully observed network to those of the subsampled network. The network metrics included degree, strength, betweenness, clustering coefficient and eigenvector centrality and the level of subsampling ranged from 0.5 to 90% of the observed sample.

For the network metrics degree and strength, the correlation was close to 0.8 even when 0.5% of the nodes were sampled. Also, the betweenness and clustering coefficient performed quite impressively at as low as 10% of the sampling level. Surprisingly, the eigenvector centrality had a minimum correlation value of 0.6 at the lowest level of sampling which was unlike the pattern we saw in the analysis of five species. This could be probably due to the fact that the sampling protocol in this case was very different from those of telemetry datasets. Most of the node-level metrics showed a very good correlation value at as low as a 10% sampling level which corresponded to 95 deer out of 951: this analysis, therefore, confirms that if we had seen only a subsample (like in the subsets observed for the five species of the main manuscript), the correlation analysis would have been useful in determining the rank order of the individual nodes with respect to the node-level metrics. This also suggests that in the future, should a researcher be motivated to use these network metrics to answer an ecological question about the fallow deer population in Phoenix Park, they can observe just 95–100 individuals over a period of time and still come to a valid conclusion about the sociality of this population (Fig. 13).

We performed the correlation analysis again, this time by sampling 500 of the total nodes (thus only half of the available sampled node population) and pretending that it was the available data to begin with. In other words, we aimed to verify how the correlation of the node-level metrics was affected if we only had half the amount of nodes to begin with. The results are depicted in Fig. 14.

The results indicate that the correlation values are still very high when the number of nodes are reduced to 500 to begin with. The pattern in these plots for each of the node-level network metrics remains identical to the previous plots except for an increased variability in the correlation values at lower levels of sampling. The strength of correlation is still good for lower levels for the network metrics of degree and clustering coefficients. This indicates that the node-level rankings with respect to different network metrics are preserved when as low as 50 individuals are observed from the population.

#### Bootstrapped confidence intervals for node-level network metrics (step 5 of the protocol)

Based on the stepwise process described in section Step 5. Bootstrapped confidence intervals for node-level network metrics, we performed node-level bootstrapping to establish confidence intervals around node-level metrics and for the Phoenix Park Fallow deer population. Given that 85% of the population had been observed and was already a part of the network structure, we predicted that the network metrics would have low uncertainty. Figure 15 depicts the results of the analysis findings. For each of the node-level network measures, the plots show narrow bands of confidence intervals around the point estimates. These findings are consistent with our expectations and support the validity of step 5 of the proposed workflow.

## Appendix 4: Node-level bootstrapping for elk, mule deer, pronghorn and roe deer

In this section, we present the results of node-level bootstrapping analysis for the networks of elk, mule deer, pronghorn and roe deer. The plots indicating the results are given in Figs. 16, 17, 18 and 19. We plotted the observed network metric values alongside the 95% confidence intervals for each node of the network. The nodes were sorted in decreasing order of the observed metric value to improve readability.

For elk and mule deer, the uncertainty in the network metric degree was lowest among all the network metrics that we tested. As the observed degree of the nodes lowered, the uncertainty in the network metric also decreased. For the network metric strength, a few nodes had a high uncertainty in the observed value as compared to other nodes in the network. This was a uniform observation across all four species and for each of the networks, the analysis allowed us to select such nodes.

For the network metric betweenness, most of the nodes had a wider lower confidence interval as compared to upper interval indicating that it was likely for them to have a lower betweenness value than their observed point estimates if a different set of individuals was sampled. Very few nodes had wider upper confidence intervals than lower. This analysis allowed us to exactly point out such nodes.

For the network metric clustering coefficient, majority of the nodes had wider lower confidence intervals than the upper. Some of the nodes with an observed clustering coefficient value of 1, had zero as the lower limit of confidence interval, indicating a very high uncertainty in the observed metric. This also indicated that in the current sample, the nodes having a value of zero as observed clustering coefficient, their value could have been much higher if some other sample was observed.

For elk and mule deer, most nodes had an observed eigenvector centrality of zero. The analysis helped identify nodes that might actually have much higher eigenvector centrality. For roe deer, only a few nodes had wide confidence intervals with an observed value of zero, suggesting that these nodes could have significantly higher eigenvector centrality compared to others if a different sample was analyzed.

Obtaining confidence intervals for the observed values of nodes in the network revealed some of the patterns that were uniform across the five species that we selected for this paper. For example, the network metric degree emerged as the most stable metric with low levels of uncertainty. These results compliment the results from the sub-sampling analysis where density emerged as the most stable metric among global network metrics. Also, the observed values for the network metrics betweenness and clustering coefficient could be much lower depending upon the choice of individuals in the sample. Therefore, care should be taken while generating inferences based on the rankings of such network metrics.

## Appendix 5: Sensitivity analysis for temporal threshold

We performed a sensitivity analysis on the choice of temporal threshold for the roe deer network. This helped us identify whether this choice significantly impacts the resultant networks and how sensitive the network is to variations in the temporal threshold.

For the observed dataset, the network structure derived remains stable and robust to the chosen temporal threshold (Fig. 20). This stability is evidenced by the minimal deviation observed in network metric values as the temporal threshold value varies. This consistency suggests that the overall structure and characteristics of the network remain largely unchanged across different temporal resolutions, indicating robustness in the network’s representation of social interactions among the observed individuals.
